# Evaluation of *Salicornia bigelovii* Germplasm for Food Use in Egypt and the United Arab Emirates Based on Agronomic Traits and Nutritional Composition

**DOI:** 10.3390/plants11192653

**Published:** 2022-10-09

**Authors:** Dionysia-Angeliki Lyra, Anitha Raman, Aly Hozayen, Rashyd Zaaboul, Fouad O. Abou-Zaid, Ahmed El-Naggar, Sherine Mansoor, Henda Mahmoudi, Khalil Ammar

**Affiliations:** 1 International Center for Biosaline Agriculture, Dubai P.O. Box 14660, United Arab Emirates; 2 International Crops Research Institute for the Semi-Arid Tropics, Patancheru 502324, India; 3 Ministry of Agriculture and Land Reclamation—The Executive Agency for the Comprehensive Development Projects, Cairo 11312, Egypt; 4 Desert Research Center, Cairo 11753, Egypt

**Keywords:** saline farming, halophytes, *Salicornia bigelovii*, germplasm evaluation, nutritional profile, desert environments, food use

## Abstract

Climate change significantly aggravates the quality of soil and water, especially in desert regions such as the United Arab Emirates (UAE) and Egypt concluding in an alarming increase in salinity in the reservoirs of the natural resources. Saline farming rises as a promising solution, utilizing low-quality water and land resources to grow salt-tolerant varieties of conventional crops and halophytes. Samphire (*Salicornia* spp.) is among the most popular multi-purpose halophytes that are locally consumed in several countries around the world as a vegetable. Six *Salicornia bigelovii* genotypes (ICBA-2, ICBA-3, ICBA-4, ICBA-8, ICBA-9, ICBA-10) were evaluated for their agronomic performance and nutritional composition in Dubai in UAE and, for the first time, at the Red Sea Governorate in Egypt in the 2019–2020 season using saline groundwater for irrigation (EC_w_ = 26 and 6.6 dS/m, respectively). ICBA-10 performed well in both locations with high green biomass and seed yield (10.9 kgm^−2^ and 116.3 gm^−2^, respectively, in UAE; 7.7 kgm^−2^ and 82.9 gm^−2^, respectively, in Egypt). ICBA-10 was, overall, also good in ion accumulation, total amino acids and unsaturated fatty acids content in both locations for shoots and seeds. Our results indicated that a lack of a drainage system and leaching fraction, the silt loam texture and the drip irrigation system might have contributed in the gradual accumulation of salts in the soil at Mubarak Valley at the end of the experiment at a higher level than ICBA. Apart from the agronomic parameters, higher salinity levels also affected ion accumulation, the amino acids and the fatty acids content for both shoots and seeds, whereas the proximate composition was affected to a lesser extent. Our findings on the high unsaturated fatty acids content under higher salinity corroborate the nutritional value of *S. bigelovii* oil. Due to its euhalophyte nature, *S. bigelovii* is a valuable source of minerals, amino acids and antioxidants that render it the most promising salt-loving plant for food use.

## 1. Introduction

The Middle East and North Africa (MENA) region is one of the most vulnerable parts of the world, combatting water scarcity and challenging distress on food production [1]. MENA is characterized by the lowest availability of water and arable land per capita in the world, threatening the food sovereignty in the region. A reduction in precipitation rates and an increase in temperature have led to prolonged droughts that cause a decline in water quality and induce salinity in the groundwater reservoirs. The United Arab Emirates (UAE) and Egypt have placed emphasis on the increase in local food production by utilizing all the available natural resources in a sustainable manner. Freshwater with Total Dissolved Solids (TDS) < 1300 ppm is very limited in UAE and accounts for 11% of the groundwater resources, out of which only 3% are found near Liwa and Al Ain, two of the most important agricultural zones in the UAE [2]. About 53% of the groundwater in this aquifer is brackish with TDS < 16,000 ppm, while 44% has high salinity TDS > 16,000 ppm and is unsuitable for any use. In Egypt, the salinity of the groundwater across the country ranges from less than 1000 to 12,000 ppm [3]. Conventional agriculture cannot be practiced using brackish groundwater unless the water is desalinated [4]. Desalination technologies are still expensive to be implemented, mainly due to massive electricity loads that are needed to remove the salts and the brine management that rises the environmental costs. However, considerable efforts are currently channeled to decrease expenses with the introduction of new methods and new energy sources [5,6]. Wherever inland desalination is not feasible, saline groundwater is the only available source to be used for irrigation purposes. Therefore, alternative new crops should be sought in order to sustain food and feed production in salinized areas since the majority of crops and forages used in modern agriculture are glycophytes (salt-sensitive) [7]. Halophytes are salt-loving plants and their growth rate is stimulated at a salinity range between 9600 and 19,200 ppm [8]. Optimal growth is attained at 3200 ppm for the monocots halophytes and between 6400 and 12,800 ppm for the dicots halophytes [9]. Halophytes possess the salt-tolerance trait that gives them the leverage to thrive in saline conditions, even up to seawater salinity, without a compromise in growth [8,10,11].

Among several halophytic plants that offer a good perspective of saline agriculture with significant economic returns is the *Salicornia* genus [12,13]. *Salicornia* spp. are annual plants. Their root system can tolerate continuous exposure to high-salinity levels, absorb salts from the medium they grow through their roots [14] and accumulate NaCl in their shoot tissues [15]. *Salicornia* is multifunctional and every part of the plant is useful [7,15,16,17,18]. Nutritional analyses have shown that *Salicornia* shoots are characterized by high protein, minerals, vitamins and polyunsaturated fatty acids (PUFA), which make it an ideal nutritional supplement [13,19,20,21,22]. Recently, efforts are deployed to initiate local *Salicornia* production and build its value chain in the UAE focusing on both fresh and processed food products [23]. 

*Salicornia bigelovii* is one of the most plausible species of the *Salicornia* genus because of the high production of biomass [16,24,25,26,27]; the protein and oil content of the seeds is similar to that of safflower 32% and 34%, respectively [7,28,29]. Research findings have shown that *S. bigelovii* biomass irrigated with seawater can be incorporated into the diets of lambs, goats and camels at an inclusion rate of 25–50% [26,30,31]. *S. bigelovii* seed meal can also be used as a potential feed source and can replace conventional seed meals in livestock diets. There have been considerable efforts toward selective breeding of *S. bigelovii* due to its high salt tolerance, its high biomass and oilseed production in desert environments and its remarkable versatility in use [29]. 

Since 2012, the International Center for Biosaline Agriculture (ICBA) is focusing on field selection of the best performing *S. bigelovii* genotypes in each cultivating season in terms of biomass and seed production. In the present study, six selected genotypes were evaluated at two locations at ICBA in the UAE and at Mubarak Valley in Marsa Alam at the Red Sea Governorate in Egypt for the season 2019–2020. It was the first time that *S. bigelovii* was introduced to the Red Sea Governorate. The objectives of the study were to: (i) evaluate the performance of six *S. bigelovii* genotypes at Mubarak Valley (the new location) compared to ICBA, (ii) identify the potential use of the *S. bigelovii* genotypes for food extracting information based on the nutritional composition (proximate composition, micronutrients, essential/non-essential amino acids and fatty acids) of shoots and seeds and (iii) identify the best *S. bigelovii* genotypes for trait(s) based on both growth and nutritional composition parameters in the two locations through multivariate analysis. 

## 2. Materials and Methods

### 2.1. Plant Material and Field Trials 

Seeds of six *S. bigelovii* genotypes (ICBA-2, ICBA-3, ICBA-4, ICBA-8, ICBA-9, ICBA-10) were sown on the 15th of November 2019 at ICBA’s experimental station (25°05′44″ N 55°23′27″ E) in Dubai UAE and on 20 December 2019 at Mubarak Valley (25°29′01″ N 34°37′29″ E) in Marsa Alam at the Red Sea Governorate in Egypt. The genotypes constituted improved germplasm after six years of selection at ICBA’s research station. ICBA-2, ICBA-3 and ICBA-4 genotypes were originally collected from salt marshes and coastal wetlands in Texas, whereas ICBA-8, ICBA-9 and ICBA-10 originated from similar coastal ecosystems in Arizona. Before sowing, the field was ploughed and organic compost was added at a rate of 20 t/ha for the trial at ICBA, whereas no compost was added in the trial at Mubarak Valley. 

The seeds/seedlings were irrigated with saline groundwater in both locations. Groundwater salinity was EC_w_ = 26 dS/m and 6.6 dS/m in Dubai and Mubarak Valley, respectively. Bubblers and drippers were installed at field trials at ICBA and Mubarak Valley, respectively, to irrigate *S. bigelovii* plants. A 30% fraction was calculated to leach the applied water and avoid salt accumulation in the root zone at ICBA, whereas no leaching fraction was applied at Mubarak Valley for *S. bigelovii* irrigation. 

**Figure 1:**
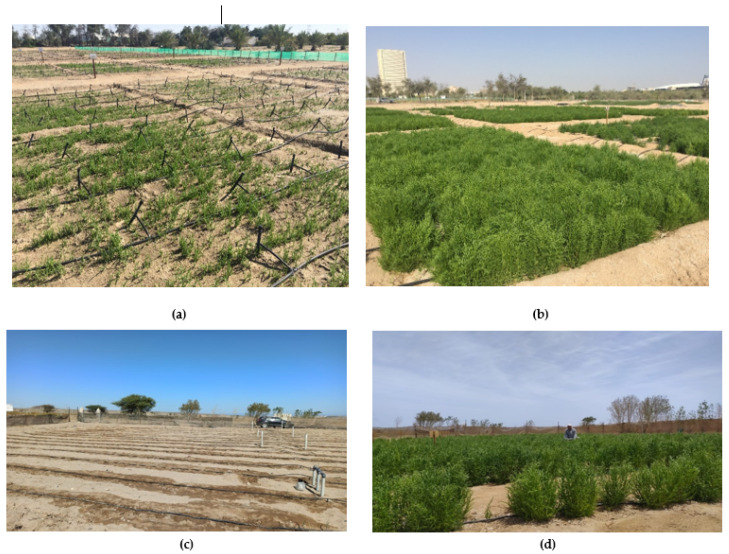
*Salicornia bigelovii* field experiments at ICBA’s experimental station at (**a**) young and (**b**) advanced vegetative stage and at Mubarak Valley in Marsa Alam at (**c**) sowing and (**d**) advanced vegetative stage.

**Figure A1:**
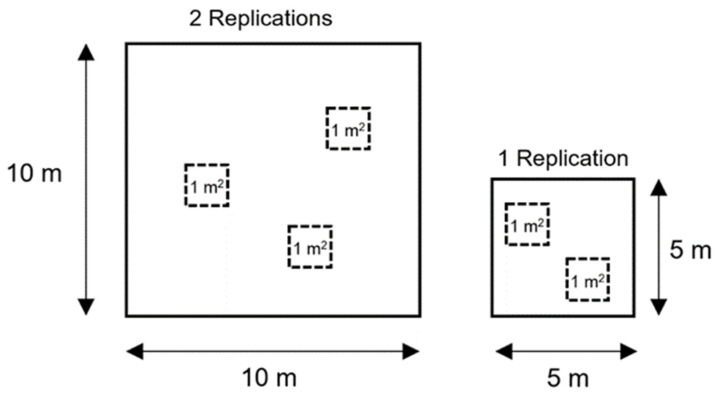
Salicornia experimental plots 10 m × 10 m (**a**) and 5 m × 5 m (**b**) at ICBA’s research station in the UAE.

**Figure A2:**
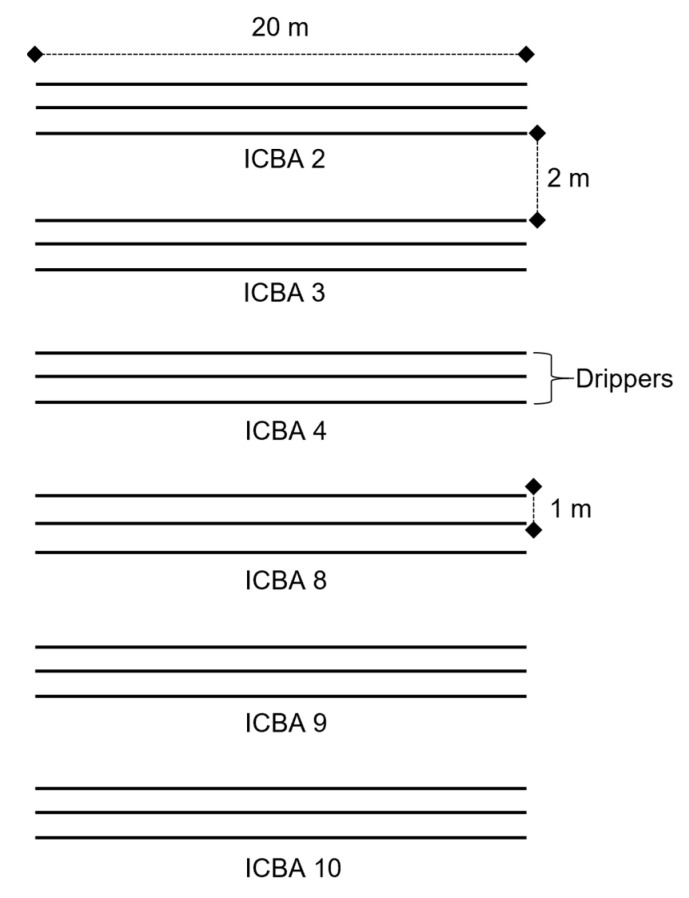
Salicornia field layout at Mubarak Valley in Marsa Alam in Egypt.

The trial was laid out in Randomized Complete Block Design (RCBD) with three replications at ICBA’s experimental station. The plot size was 10 m × 10 m in two replications and 5 m × 5 m in the third (Figure A1). A row-to-row distance of 50 cm was maintained (20 rows for 10 m × 10 m and 10 rows for 5 m × 5 m plots). Three lines of 20 m for each *S. bigelovii* genotype were established at Mubarak Valley in Marsa Alam (Figure A2) with a row spacing of 1 m and genotype-to-genotype spacing of 2 m. Each line corresponded to one replication per genotype. The seeds were sown at a rate of 0.5 g/m^2^ in both ICBA and Mubarak Valley. The sowing was conducted along the rows in continuous sequence at ICBA whereas plant-to-plant spacing was 20 cm at Mubarak Valley. Photographs of the *S. bigelovii* field experiments at ICBA and Mubarak Valley are presented in Figure 1.

### 2.2. Plot Data 

The emergence of *S. bigelovii* seedlings was monitored at 19 and 48 days after sowing (DAS) at ICBA and at 21 and 49 DAS at Mubarak Valley (Table 1). The percentage was calculated based on visual inspection by scanning the coverage of the cultivated area (plots at ICBA and rows at Mubarak Valley) by *S. bigelovii* seedlings. 

The plots were harvested at an advanced vegetative stage on 7 and 10 June at ICBA and Mubarak Valley, respectively. *S. bigelovii* plants were collected from three quadrats 1 m × 1 m (1 m^2^) from the two replications (plot size 10 m × 10 m) and from 2 quadrats (1 m^2^) from the third replication (plot size 5 m × 5 m) at ICBA (Figure A1). Thus, eight quadrats of 1 m^2^ were evaluated from all three replications. The total number of plants per quadrat, the plant height of three plants per quadrat and the green biomass/m^2^ of all the plants contained in the quadrat were measured. The same measurements were taken at Mubarak Valley from 4 running meters along the row (sample size 4 × 0.5 = 2 m^2^). One measurement was taken per row (per replication) for the number of plants and green biomass yield (GBY), whereas three individual plants per replication were measured for plant height. At maturity, seeds were harvested in late August and early September at ICBA and Mubarak Valley, respectively. The sampling protocol for the seed yield (SY) was similar to the one followed for the previous measurements (8 quadrats of 1 m^2^ at ICBA and 3 replications of 2 m^2^ at Mubarak Valley). 

### 2.3. Climatic Data 

Climatic data such as temperature (minimum, maximum, average, dew point) and precipitation were extracted for both locations from ERA 5-Land [32] (https://cds.climate.copernicus.eu/#!/home, accessed on 17 April 2022). This dataset is a global gridded climatic data on a regular latitude-longitude grid of 0.1° × 0.1° resolution. Relative humidity was calculated from 2m temperature and dew point daily data using the Formula (1):

RH = 100 × E/Es (1)

where E and Es are actual vapor pressure and saturated vapor pressure, respectively. E can be calculated by Formula (2) whereas Es by Formula (3):

E = 0.611 × exp(5423 × (1/273 − 1/Td)) (2)

Es = 0.611 × exp(5423 × (1/273 − 1/T)) (3)

where T and Td are the 2m temperature and dew point in Kelvin, respectively.

Both ICBA’s experimental station in Dubai and Mubarak Valley in Marsa Alam are located at the same geographical latitude (25.1 N for ICBA and 25.4 N for Marsa Alam) and at a close distance from the sea (15 Km for ICBA and 5 Km for Marsa Alam). Therefore, both Dubai and Marsa Alam are characterized by a hot and desert climate, with very mild winters and very hot and sunny summers. In Marsa Alam, the climate is highly influenced by the sea, whereas in Dubai the climate is highly impacted by the desert. The differences between the maximum and minimum temperature were almost uniform throughout the year (around 7 to 8 degrees difference between night and day temperatures) in Marsa Alam, whereas these differences were larger (around 10 to 16 degrees difference between night and day temperatures) in Dubai. Table 2 summarizes the monthly means of temperature (Tmin and Tmax), relative humidity (RH) and rainfall over the period 2000–2020. Minimum temperatures (night-time) were quasi-identical in both locations while maximum temperatures (daytime) were higher in Dubai than in Mubarak Valley, especially during summertime where differences between the two locations reached 7 to 8 degrees. Rainfall and relative humidity showed big differences between the two locations. Mubarak Valley exhibited drier climatic conditions than Dubai. 

The same climatic parameters were explored for the growing season of *S. bigelovii* from 1 November 2019 till the end of October 2020 in both areas (Section 3.1). 

### 2.4. Soil and Water Analysis 

Soil samples were collected from two depths (0–15 top surface soil and 15–45 subsurface soil) before and after the experiments. Soil paste extract was prepared using the Buchner method [33]. Based on the procedure, 10 g of <2-mm air-dried soil was placed in a 125-mL Erlenmeyer flask and mixed with 1N NH4OAc. The mixture was then transferred to a 5.5-cm Buchner funnel fitted with No 42 Whatman filter paper and connected to a 250 mL suction flask. The extract was then poured into a 250 mL measuring flask for the determination of the soil pH and electrical conductivity (EC_e_) [34]. Exchangeable sodium (Na^+^) and potassium (K^+^) were determined in the paste extract using Flame Photometer Jenway model PFP7. Calcium (Ca^2+^) and magnesium (Mg^2+^) were determined in the extract by the EDTA titrimetric method using an Erichrome Back T (EBT) indicator [33]. Soil bicarbonate (HCO_3_^−^) was measured in the saturated paste extract by titration with 0.025 N H_2_SO_4_ [35] and chloride (Cl^−^) using the titration with Silver Nitrate [36]. Sulfate (SO_4_^2^^−^) was estimated by subtraction. Soil inorganic nitrogen (N) was measured using the Kjeldhal method in a 2M KCl extract [37]. Soil available phosphorus (P) and potassium (K) were measured using an ammonium bicarbonate-diethylenetriaminepentaacetic acid (AB-DTPA) solution [38]. The extracted phosphorus (P) was determined calorimetrically using the molybdate-ascorbic acid method. Soil micronutrients (Cu, Fe, Mn and Zn) were determined using ICP in the AB-DTPA extract. Soil texture (particle size distribution) was determined by the hydrometer method by measuring the fractions of sand, silt and clay [39]. 

In irrigation water, EC_w_ and pH were measured using the EC and pH meters [40]. The concentrations of soluble Na^+^ and K^+^ were assessed via flame photometry (Jenway, UK) [41]. Ca^2+^ and Mg^2+^ were determined by the Versenate method [42]. SO_4_^2−^ content of the water samples was gravimetrically estimated using a BaCl_2_ solution [43], while Cl^−^ concentration was assessed by a titration method using AgNO_3_ [44]. HCO_3_^−^ was also determined by the titration method [45]. Micronutrients were determined as per [46]. Nitrate (NO_3_^−^) was determined by a spectrophotometer [47]. Ammonium (NH_4_^+^) and PO_4_^3−^ were measured in water using DR 3900 HACH spectrophotometer [48].

### 2.5. Analysis of the Chemical Composition of Shoots and Seeds

The nutritional profile of shoots and seeds of all six *S. bigelovii* genotypes was studied for both locations by analyzing the proximate composition, micronutrients, amino acids and fatty acids content. Sampling was performed randomly in the replications (three plots at ICBA and three rows at Mubarak Valley per genotype) and one composite sample of fresh tips from different plants per genotype was prepared for the nutritional analyses in both locations. A similar approach was followed for the preparation of the composite samples of seeds from all three replications per genotype at the two locations. Thus, six samples of shoots and six samples of seeds were analyzed in total per location. The fresh tips (10-15 cm) were cut with scissors approximately three and a half months after sowing, on the 3rd of March at ICBA and on the 5th of April at Mubarak Valley, whereas seed samples were prepared after the final harvest of the dry biomass for the seeds collection. Seed samples were prepared by removing them through mechanical separation. The seeds were washed with deionized water and then dried with a towel, in order to remove the external excess of salt. Nutritional analyses followed the methodology of the Association of Official Analytical Chemicals [49]. Water content (WC) was determined by the difference between fresh matter and dry matter; fat (FAT) was determined by the Soxhlet extraction method; protein (PS) was measured by the Kjeldahl method; crude fiber (CF) content was determined using the neutral detergent reagent method [21]; and total carbohydrate (CHO) content was estimated by the difference between 100 and the sum of the percentages of moisture, protein, total lipid and ash contents [21]. Total ash content (Ash) was analyzed after burning the plants in a muffle furnace. The micronutrients sodium (Na^+^), potassium (K^+^), magnesium (Mg^2+^), manganese (Mn^2+^), calcium (Ca^2+^), phosphorus (P^3−^), iron (Fe^2+^) and zinc (Zn^2+^), were analyzed using the Inductive Coupled Plasma (ICP) spectrometer and atomic absorption [50]. The ratio Na^+^/K^+^ was calculated by dividing the corresponding values. Τhe vitamins C (VIT C), B1 (VIT B1) and B2 (VIT B2) were measured based on high-performance liquid chromatography (HPLC) analysis. Essential and non-essential amino acids were determined by liquid chromatography–mass spectrometry *(*LC*–*MS/MS*)* and an amino acid analyzer. The total amino acids content (TAA) was the sum of all the essential and non-essential amino acid values. 

The fat extracted from seeds was further analyzed for the fatty acids composition using gas–liquid chromatography (GLC) and gas chromatography mass spectrometry methods [51,52]. Identification of each fatty acid was conducted using the equivalent chain lengths and laboratory standards. The total unsaturated fatty acids (USFA) were the sum of mono- (MUFA) and poly-unsaturated fatty acids (PUFA) content. The total saturated fatty acids (SAFA) content was calculated as the sum of the saturated fatty acids content. The ratios USFA/SAFA and Oleic/Linoleic were calculated by dividing the corresponding values.

The abbreviations of the nutritional parameters analyzed for *S. bigelovii* shoots and seeds are presented in Table 3. More information about the methodologies applied and equipment used for the chemical analyses is provided in the Supplementary Material. The values derived from the nutritional analyses were expressed on fresh (FW) and dry weight (DW) for shoots and seeds, respectively. 

### 2.6. Statistical Analysis 

Data from the trial at ICBA was analyzed using PBtools version 1.4. (2014). Genotype means were estimated using a mixed model, taking genotypes as fixed and replicates as random. For the trial at Mubarak Valley, simple average and standard error values were calculated for all agronomic traits measured. The data of nutritional traits together with GBY values for the shoots and SY for the seeds were subject to principal component analysis (PCA) using the R software to investigate: (i) correlations among the nutritional traits and (ii) commonalities and differences among *S. bigelovii* genotypes. The correlation matrix between the traits was the driver for PCA. To overcome the problem of skewness due to the units of measurement, standardization was conducted and the data were converted to a mean of 0 and standard deviation of 1. The first two principal components (Dim 1 and Dim 2) captured most of the variation in the data. The PCA scores for the traits were the weights that defined the importance of each trait and genotype to the respective PC. PC biplots derived from PCA visualizing the results. The biplot combined both the PC scores for the genotypes and the loading vectors of the traits in a single biplot display. 

## 3. Results

### 3.1. Climatic Data 

**Figure 2:**
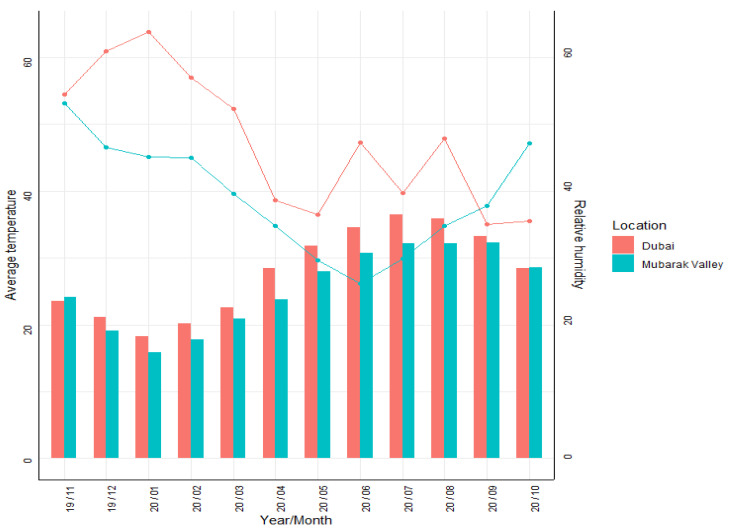
Average monthly temperature and relative humidity from November 2019 to October 2020 at ICBA in Dubai and Mubarak Valley in Marsa Alam. Data source: ERA5.

During the growing season from November 2019 to October 2020, the weather in Mubarak Valley was colder and drier than in Dubai. The differences in monthly average temperature between the two locations ranged from 0.2 to 4.8 degrees in winter and summer, respectively (Figure 2). Dubai received 145 mm of rainfall for the entire growing season with some rare and intense rainfall in November 2019 (16 mm) and January 2020 (73 mm)**,** whereas Marsa Alam recorded just 2.8 mm of rainfall for the same period. 

### 3.2. Water and Soil Analyses

#### 3.2.1. Water analysis 

Salicornia was irrigated with saline groundwater at both sites. The salinity of groundwater at ICBA’s site was four folds higher (EC_w_ = 26.0 dS/m) than the water salinity at Mubarak Valley (EC_w_ = 6.6 dS/m) (Table 4). Water was slightly alkaline in both locations with similar pH values (7.4). In general, the concentrations of all tested parameters were higher at ICBA’s site compared to Mubarak Valley, except for HCO_3_^−^ and NH_4_^+^ which were lower. The concentrations of anions Cl^−^ and SO_4_^2−^ were almost four and six times higher at ICBA’s experimental station compared to Mubarak Valley, respectively. Cl^−^ concentration (204.0 meql^−1^) at ICBA’s research station exceeded the threshold of 10 meql^−1^ above which the water can cause severe problems to conventional crops [53]. Ca^2+^, Mg^2+^, Na^+^, K^+^ and NO_3_^−^concentrations were higher at ICBA’s site compared to Mubarak Valley. This high content could also affect the growth of conventional crops [54]. The rest of the elements B, Cu, Fe, Mn, Zn and PO_4_^3−^ were characterized by either negligible or very low concentrations.

#### 3.2.2. Soil Analysis 

The soil analysis revealed significant differences in the soil properties between ICBA and Mubarak Valley (Table 5). The soil at ICBA is characterized as sandy since it is mostly comprised of sand (96.5 percent) whereas the soil in Marsa Alam is characterized as silt loam, and it comprises of 53 and 38.5 percent of silt and sand, respectively. In both sites, the soil salinity was highly increased in both surface and subsurface soil after applying saline water for irrigation. The surface and subsurface soil at the end of the experiment at ICBA was characterized by EC_e_ = 12.3 and 14.5 dS/m, respectively, whereas at Mubarak Valley was characterized by much higher salinity EC_e_ = 28.2 and 8.9 dS/m, respectively. Soil salinity was more pronounced in the surface soil (top 15cm) compared to 15–45 cm layer at Mubarak Valley, due to the high-evaporation rate [55]. SO_4_^2−^, Cl^−^, Na^+^, Mg^2+^ and Ca^2+^ content significantly increased after applying saline water for irrigation in both locations, whereas the increase for K^+^ and HCO_3_^-^ was lower. The concentrations of all the available nutrients increased after applying water of higher salinity at ICBA’s experimental station, whereas they decreased at Mubarak Valley. 

### 3.3. Salicornia bigelovii Agronomic Measurements

#### 3.3.1. Seedlings’ Emergence

**Figure 3:**
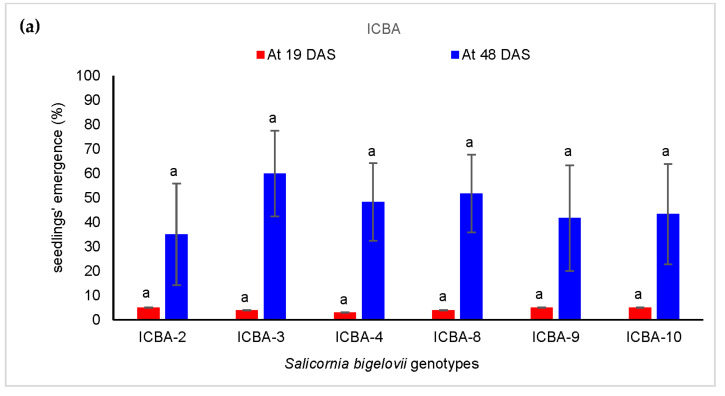

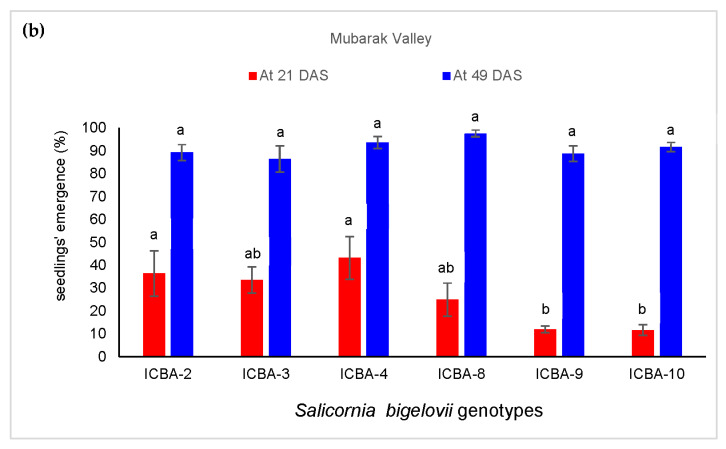
Seedlings’ emergence (%) of *Salicornia bigelovii* after (**a**) 19 and 48 days from sowing at ICBA and (**b**) 21 and 49 days from sowing at Mubarak Valley.

After 19 DAS, not more than 5 percent of seedlings emerged at ICBA for all genotypes, whereas the emergence of seedlings ranged from 12 (ICBA-9 genotype) to 43 (ICBA-4) percent after 21 DAS at Mubarak Valley (Figure 3). After 48 DAS at ICBA, more than 35 percent of the seedlings (ICBA-2) emerged up to 60 percent (ICBA-3). Higher values of emergence were observed for all genotypes at Mubarak Valley, and these ranged between 86 (ICBA-3) and 97 (ICBA-8) percent after 49 DAS. This finding implied that the seedlings’ emergence phase was completed earlier under low salinity at Mubarak Valley compared to higher saline conditions at ICBA’s experimental station. In the current study, seedlings’ emergence was not statistically different among genotypes at ICBA’s experimental station for both observation dates (19 and 48 DAS) nor for the genotypes evaluated at Mubarak Valley after being measured at 49 DAS. Fewer seedlings of ICBA- 9 and ICBA-10 emerged at 21 DAS compared to the rest of genotypes at Mubarak Valley and the values were statistically significantly different compared to the rest. After 49 DAS, more seedlings of ICBA-9 and ICBA-10 genotypes emerged similarly to the rest of the genotypes tested. 

#### 3.3.2. Growth and Yield Parameters 

For the trial at ICBA, no statistically significant differences were observed in a number of plants per m^2^, plant height and seed yield, whereas there were significant differences between the genotypes for green biomass (Table 6). On the contrary, the genotypes at Mubarak Valley showed significant differences for all parameters except the number of plants per m^2^. ICBA-10 yielded well in both sites, although the plant number for this genotype was the lowest at ICBA (23 plants per m^2^ on average) and highest at Mubarak Valley (45 plants per m^2^ on average). ICBA-8, ICBA-10 and ICBA-3 were the best for green biomass at ICBA while ICBA-10, ICBA-9 and ICBA-2 were the best for green biomass at Mubarak Valley. ICBA-10, ICBA-4 and ICBA-8 were characterized by the highest seed yield values at ICBA, whereas ICBA-8, ICBA-4 and ICBA-10 produced more seed at Mubarak Valley. The plants were in general taller at Mubarak Valley compared to ICBA. ICBA-8 and ICBA-10 generally outperformed other genotypes at both sites. 

### 3.4. Evaluation of the Nutritional Composition of Shoots and Seeds through PCA

#### 3.4.1. Nutritional Analysis of Shoots 

Overall, no major differences were observed in the proximate composition of shoots between the two locations (Table A1). WC represented the largest single content (89% on average) for all genotypes in both locations. CHO (5.15 percent on average) and ash (4.35 percent on average) were the second and third parameters, respectively, followed by PS (1.05 percent on average), fat (0.2 percent on average) and CF (0.15 percent on average) for all genotypes in both locations. Na^+^ was more than double in the shoots at Mubarak Valley (3429 mg/100 g) compared to the shoots at ICBA (1538 mg/100 g). Opposite results were observed for K^+^ which were characterized by higher values for ICBA (281.7 mg/100 g) compared to Mubarak Valley (162.5 mg/100 g). This also explains the increased fourfold Na^+^/K^+^ ratio at Mubarak Valley compared to the Na^+^/K^+^ ratio for ICBA. The same trend as K^+^ was noted for Ca^2+^, Mg^2+^, P^3−^, whereas Zn^2+^, Mn^2+^ and Fe^2+^ were characterized by similar values in both locations. VIT B1 and B2 were traced in the shoots at Mubarak Valley, whereas there was almost no detection of the two vitamins for plants grown at ICBA. In contrast, VIT C content was much higher in the shoots at ICBA compared to Mubarak Valley. At ICBA, ICBA-2 and ICBA-10 genotypes were characterized by the highest TAA content, 1116.9 and 1268.9 mg/100 g, respectively, compared to the rest of the genotypes, whereas 783.0 and 781.0 mg/100 g were the highest TAA concentrations measured for ICBA-4 and ICBA-8 at Mubarak Valley, respectively (Table A2). Glu was the amino acid with the highest concentration more than 100 mg/100 g FW for the majority of genotypes in both locations. 

**Figure 4:**
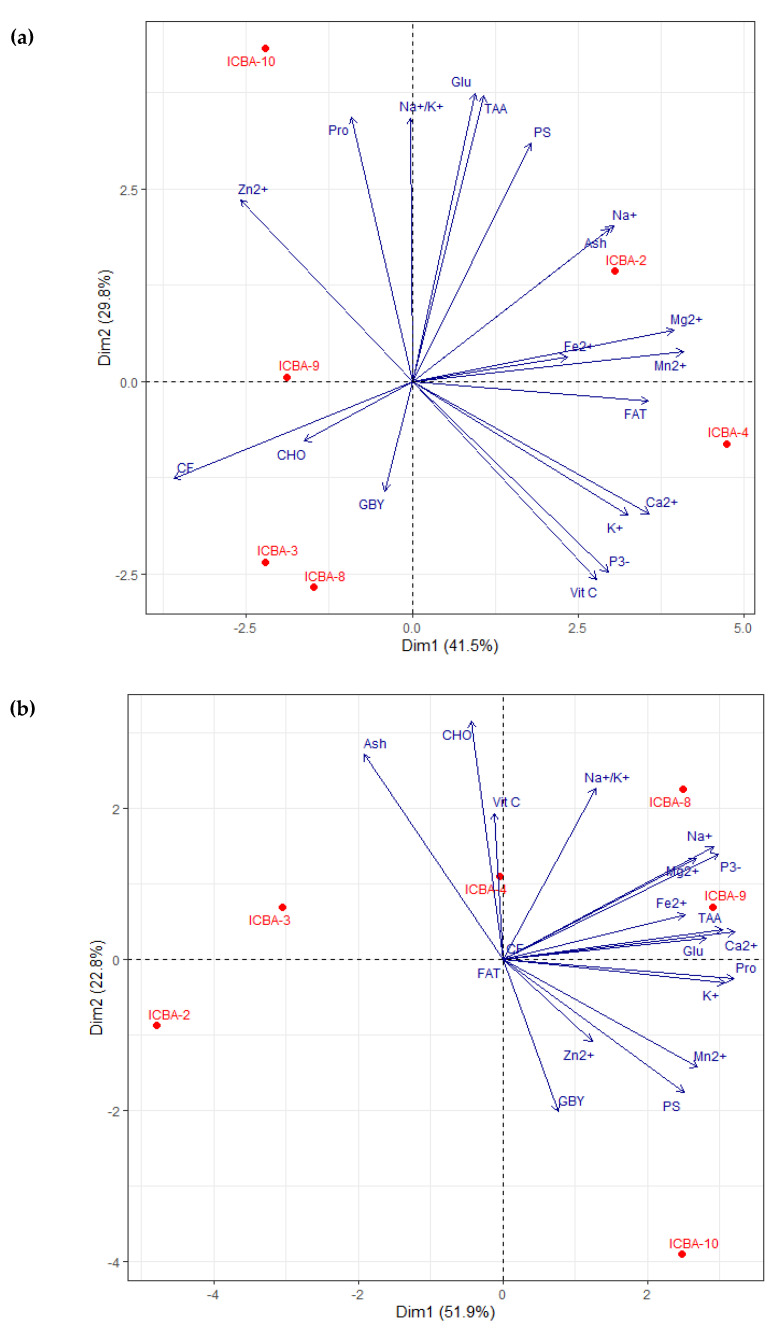
Plot of Principal Component Analysis (PCA) for the green biomass yield, proximate composition, micronutrients and amino acids for six *S. bigelovii* genotypes at: (**a**) ICBA and (**b**) Mubarak Valley for the first two dimensions. See Table A7 for eigenvector values of each parameter for PCA dimension 1 (Dim1) and 2 (Dim2).

Based on the PCA plot for ICBA, the first two PC dimensions for the shoots explained together 71.3 and 74.7 percent of the total variation in the data obtained from ICBA (Figure 4a) and Mubarak Valley (Figure 4b), respectively. For the trials at ICBA, contrasts among the genotypes were clearly brought out at each quadrant. The traits that had a big influence on placing ICBA-3 and ICBA-8 on the left-hand side and ICBA-4 on the right-hand side can be identified by looking at their corresponding eigen values on PC1 and PC2 (Table A7). ICBA-4 was good for FAT, K^+^, Ca^2+^, P^3−^ and VIT C (positive PC1 and negative PC2 scores). ICBA-3 and ICBA-8 were high in CHO, CF and GBY (negative PC1 and PC2 scores). ICBA-2 was rich in PS, Ash, Na^+^, Mg^2+^, Fe^2+^, Mn^2+^, Glu and TAA (positive PC1 and PC2 scores). ICBA-10 was high in Na^+^/K^+^, Pro and Zn^2+^ (negative PC1 and positive PC2 scores). No trait was identified close to ICBA-9. 

For the experiment at Mubarak Valley, a different pattern was observed for the genotypes. ICBA-8 and ICBA-9 were clustered in the same quadrant and were good for Na^+^, Na^+^/K^+^, Ca^2+^, Mg^2+^, Fe^2+^, P^3−^, Glu and TAA (positive PC1 and PC2 scores) (Table A7). ICBA-3 and ICBA-4 did well for VIT C, CHO and Ash (negative PC1 and positive PC2 scores). ICBA-10 was high in GBY, PS, K^+^, Mn^2+^, Zn^2+^ and Pro (positive PC1 and negative PC2 scores). ICBA-2 was not specifically good for any of the traits. 

Comparing the two sites for the shoot traits, ICBA-3 was consistently good for CHO; ICBA-4 for VIT C; and ICBA-10 for Zn^2+^ and Pro. This classification across two sites is only an initial orientation. Repeated tests are required to assess the genotypes for stability across years since this analysis was based on one season only.

#### 3.4.2. Nutritional Analysis of Seeds 

CHO was characterized by the biggest share in the proximate composition of seeds and the content was higher at ICBA (47.4 percent on average) compared to Mubarak Valley (37.6 percent on average) (Table A3). After CHO, FAT and PS content were the parameters with relatively big values in the proximate composition 19.1 and 18.1 percent (on average for all genotypes), respectively, at ICBA and 20.1 and 22.8 percent (on average for all genotypes), respectively, at Mubarak Valley. Then CF, Ash and WC followed with percentages of 8, 4.2 and 3.4 (on average) at ICBA and 9.4, 6.2 and 4.1 (on average) at Mubarak Valley, respectively. Regarding micronutrients, *S. bigelovii* genotypes grown at ICBA were characterized by higher Na^+^, Ca^2+^, P^3−^, VIT C and VIT B1 content and lower concentrations of K^+^, Mg^2+^, Fe^2+^, Mn^2+^, Zn^2+^ and VIT B2 compared to the ones obtained at Mubarak Valley. It is worth noticing that the variability among the genotypes for most of the nutritional attributes was higher for the seeds as compared to the shoots at each location. 

In general, higher concentrations of essential and non-essential amino acids were measured for the seeds of the six genotypes cultivated at Mubarak Valley compared to ICBA (Table A4). ICBA-2 and ICBA-8 at ICBA trials were characterized by the highest TAA, 10,720 and 10,480 mg/100 g, respectively, whereas at Mubarak Valley, ICBA-4 and ICBA-9 had the highest TAA content of 15,670 and 20,256 and mg/100 g, respectively. Glu was the amino acid with the highest concentration more than 2000 mg/100 g in both locations. Asp, Cys and Gly were also characterized by higher concentrations (>1000 mg/100 g) for some genotypes at Mubarak Valley. 

All genotypes in both locations had LA (C18:2 ω6) in the highest concentration ranging from 39.1 to 62.2 percent (Table A5). For the seeds collected at ICBA, OA (C18:1 ω9) and PA (C16:0) were in the second (18.2 percent on average) and third (13.5 percent on average) position, respectively, followed by SA (C18:0) (5.8 percent on average) and ALA (C18:3 ω3) (1.9 percent on average). At Mubarak Valley, after LA (C18:2 ω6) (50.7 percent on average), PA (C16:0) (23.9 percent on average), OA (C18:1 ω9) (6.9 percent on average), SA (C18:0) (3.6 percent on average) and MA (C14:0) (3.2 percent on average) followed. Overall, the seeds of all genotypes at ICBA were characterized by higher values for USFA, USFA/SAFA and OA (C18:1 ω9)/LA (C18:2 ω6) ratio and lower values for SAFA compared to Mubarak Valley. 

**Figure 5:**
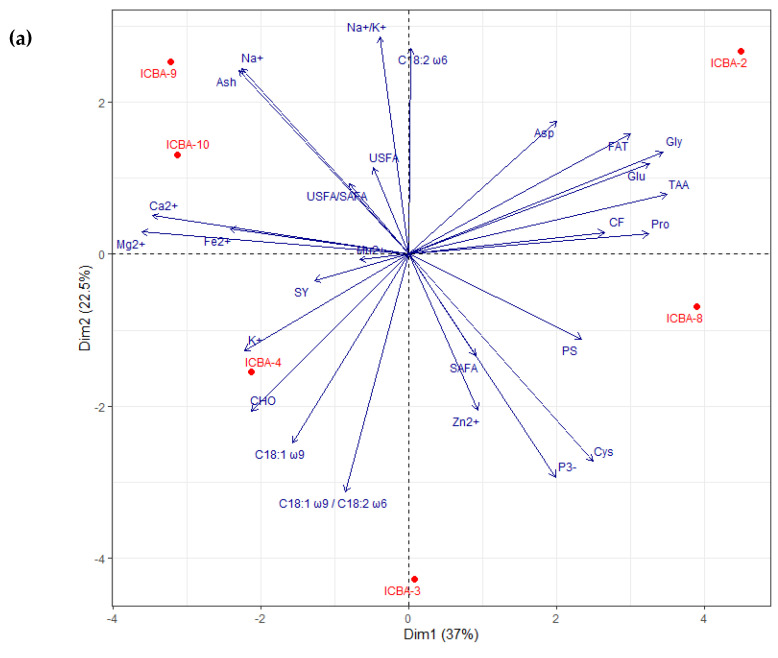

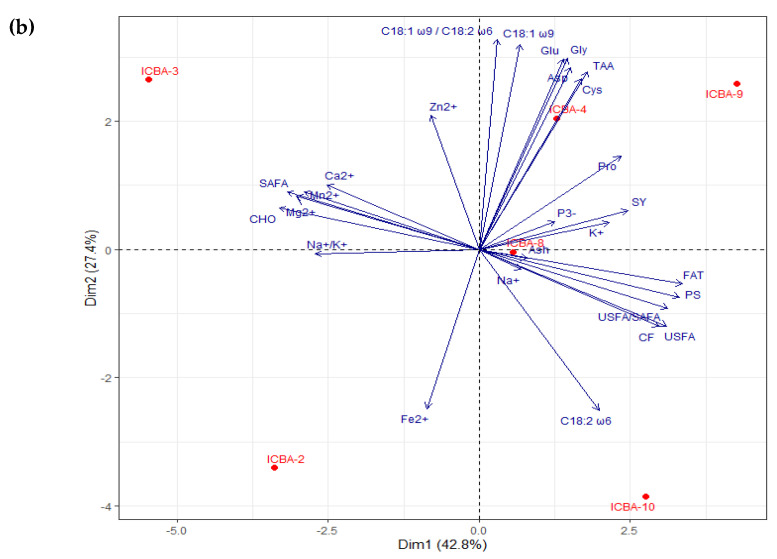
Plot of Principal Component Analysis (PCA) for seed yield, proximate composition, micronutrients, amino acids and fatty acids for six *S. bigelovii* genotypes at: (**a**) ICBA and (**b**) Mubarak Valley for the first two dimensions See Table A8 for eigenvector values of each parameter for PCA dimension 1 (Dim1) and 2 (Dim2).

The first two PCA dimensions captured 59.5 percent of the total variation in the data for the seeds obtained from ICBA (Figure 5a). ICBA-9 and ICBA-10 were grouped in the upper left quadrant of the graph and did well for Ash, Na^+^, Na^+^/K^+^, Ca^2+^, Mg^2+^, Fe^2+^, USFA and USFA/SAFA ratio (Table A8). ICBA-2 was a good performer for FAT, CF, Asp, Glu, Gly, Pro, TAA and LA (C18:2 ω6). ICBA-8 and ICBA-3 did well for PS, Zn^2+^, P^3−^, Cys and SAFA. ICBA-4 was high in SY, CHO, K^+^, Mn^2+^, OA (C18:1 ω9) and OA (C18:1 ω9)/ LA (C18:2 ω6) ratio. For the seed data from Mubarak Valley, the first two principal components accounted for 70.2 percent of the variation (Figure 5b). ICBA-4 and ICBA-9 performed well for Asp, Cys, Glu, Gly, Pro, TAA, OA (C18:1 ω9) and OA (C18:1 ω9)/ LA (C18:2 ω6) ratio. ICBA-10 was good for FAT, PS, CF, LA (C18:2 ω6), USFA and USFA/SAFA ratio. ICBA-3 did well for CHO, Ca^2+^, Mg^2+^, Mn^2+^, Zn^2+^ and SAFA and ICBA-2 was good for Fe^2+^ and Na^+^/K^+^. ICBA-8 scored zero for Dim 2 (Y axis) and was positioned on the right side of the X axis between ICBA-4, ICBA-9 and ICBA-10. ICBA-8 was good for SY, Ash, Na^+^, K^+^ and P^3−^. Apart from these parameters, it is also doing well for those that are falling in the quadrants in the right side; however, with mediocre values compared to IBA-4, ICBA-9 and ICBA-10. 

Comparing the two sites for the seed traits, ICBA-3 was consistently good for Zn^2+^and SAFA; ICBA-4 for OA (C18:1 ω9) and OA (C18:1 ω9)/ LA (C18:2 ω6) ratio; and ICBA-10 for USFA and USFA/SAFA ratio. Repeated tests are required across years for stability assessments since this classification across two sites was based on one season only.

#### 3.4.3. Fatty Acids Content in Shoots and Seeds 

When comparing the fatty acids content between shoots and seeds at Mubarak Valley (Table A6), it is evident that LA (C18:2 ω6) was detected in both parts of the plant; however, in almost 30 percent lesser amount in shoots. ALA (C18:3 ω3) was measured only in the shoots, whereas PA (C16:0) was detected in both shoots and seeds at similar amounts. USFA and SAFA in shoots were 54.8 and 27.1 percent, whereas in seeds were 63 and 37.6 percent, respectively. Overall, *S. bigelovii* shoots were characterized by a more diversified range of both USFA and SAFA compared to seeds. 

## 4. Discussion

### 4.1. Climatic Conditions 

*S. bigelovii* is native to the coastal areas in Southern United States and Mexico [22]. UAE, Egypt, Southern United States and Mexico are all located at the borders between the tropical and the sub-tropical zones. Thus, it makes sense that areas within this geographical zone are explored for *S. bigelovii* cultivation. Actually, Marsa Alam and Dubai are almost located on the same latitude 25N. The climatic context in Marsa Alam and Dubai is ideal for good growth of *S. bigelovii,* since both are characterized as hot and dry locations. The agronomic performance and nutrition profile of six *S. bigelovii* genotypes was evaluated for the first time at the Red Sea region. 

### 4.2. Water and Soil Analysis

Due to prolonged droughts, the increasing salinity of the groundwater at Mubarak Valley has impeded the proper growth of vegetables traditionally grown in the area. The groundwater salinity at Mubarak Valley was 6.6 dS/m, whereas at ICBA it was four folds higher 26.0 dS/m. Alternative crops that can overcome such high saline conditions are sought. Multipurpose halophytes, such as Salicornia, with promising farming potential in hot and dry areas that can be irrigated with saline groundwater inland and with seawater in the coastal areas constitute good candidates [12,56,57,58]. In both sites, the salinity increased in the surface and subsurface soil after applying saline water for irrigation. The salinity at the topsoil at the end of the field experiment at Mubarak Valley was almost two times higher than the salinity of the topsoil measured at ICBA, although the salinity of the groundwater applied was much lower at Mubarak Valley. At ICBA, there was an effective drainage system constructed that helped to leach the salts, but this was not the case at the Mubarak Valley. In addition, a leaching fraction was applied at ICBA but not at Mubarak Valley. The increase in salinity in the latter region could be also attributed to the silt loam texture of the soil (medium-size particles) that contributed in retaining more salts in the upper soil layer, compared to the sandy soil (large-size particles) at ICBA where most of the water was leached. 

### 4.3. S. bigelovii Seedlings’ Emergence

Germination and seedlings’ emergence constitute the most critical stages in the life cycle of halophytes since they define the survival of the species and their good establishment in the local environment [59]. The seedlings started emerging two weeks after sowing at Mubarak Valley, whereas the seedlings at ICBA appeared three to four weeks after sowing (data not shown). ICBA-2 and ICBA-8 were the first genotypes to emerge at Mubarak Valley, whereas ICBA-3 and ICBA-8 seedlings appeared first at ICBA (data not shown). At Mubarak Valley, the seedlings emerged to a higher extent (more than 86%) than at ICBA (less than 60%) almost 49 days after sowing. This could be attributed to the fact that the salinity of the water used for irrigation was much lower in the former (6.6 dS/m) compared to the latter (26.0 dS/m) location. 

Under higher salinity levels, the water potential of the medium is reduced; thus, the water uptake by the imbibed seeds is impeded which results in reduced germination [13]. The sensitivity of halophytic seeds to hyper-saline conditions (between 13 to 38 ppt) was also noted in another study [60]. Apart from salinity, temperature and their interaction also play a key role in the germination rate of *Salicornia* spp. and highly impact seedlings’ emergence [61]. In our work, the average temperature did not differentiate much between the two locations in November and December (Figure 2); thus, most possibly, the main factor that played a crucial role in the seedlings’ emergence was the salinity level of the irrigation water.

Differences among *S. bigelovii* genotypes were revealed especially when higher salinity was applied at ICBA. Intraspecific variability is commonly observed at the seedlings stage when ecotypes within a species are exposed to higher salinity levels, reflecting the highest degree of salinity tolerance [57,62,63,64]. Saline water treatments above 50% of seawater concentrations applied on seeds of *Salicornia persica* and *Sarcocornia fruticosa* ecotypes revealed higher variability of the seedlings compared to lower salinity levels [21]. 

### 4.4. S. bigelovii Growth Parameters 

The six *S. bigelovii* genotypes evaluated in the current study constituted a selection of the best-performing ones out of a bigger pool of 46 after six years of assessment at ICBA’s research station. Their adaptability to a new location at Mubarak Valley in Egypt was investigated. The overall number of plants per m^2^ was higher at ICBA (51 plants per m^2^ on an average) compared to Mubarak Valley (37 plants per m^2^) probably because of the bubblers irrigation system applied and continuous sowing along the line. Bubbler irrigation is mostly used for plant species for which water requirement is higher and it is recommended in situations where salinity levels of the irrigation water are also higher [65]. *S. bigelovii* typically grows in coastal tidal marshlands and optimal irrigation was applied to maintain the humidity simulating its floodable habitat [66]. Bubblers cover a much bigger radius of the irrigated surface that might have triggered more *S. bigelovii* seeds to germinate and seedlings to emerge. ICBA-2, ICBA-9 and ICBA-10 produced the highest green biomass (more than 7.4 kgm^−2^) whereas ICBA-8 had the highest seed yield (91.4 gm^−2^) at Mubarak Valley. These yields were lower than the ones obtained at ICBA most probably due to the lower salinity of the water used for irrigation at Mubarak Valley. Salinity around 20–25 dS/m constitutes the ideal level for *S. bigelovii* and other halophytes to obtain optimum growth [7,11,67,68]. At ICBA, ICBA-8 and ICBA-10 demonstrated the highest green biomass and seed yield (Table 6). The performances of these genotypes were on par with the best performers identified in other trials at ICBA tested under similar groundwater salinity (20 dS/m) levels [57]. Although ICBA-3 was promising for biomass, seed yield was low. Within-genotype variability was high in both locations for the majority of characteristics. This variability could be attributed to the outcrossing behavior of the species [29]. 

### 4.5. Assessment of the Nutritional Profile of S. bigelovii Shoots 

The pattern observed in the proximate composition for the shoots was in the following order WC > CHO > Ash > PS > FAT > CF and it was similar to previous studies [22,26,69]. PS in *S. bigelovii* shoots (approx. 1% in the current study) is similar to lettuce (1.4%) and cabbage (1.3%) [22]. CHO are synthesized and accumulated in halophytes as compatible solutes as a response to the plants under salt stress conditions [70]. The CF content was characterized by low values (less than 1%) similar to other studies [22]. In contrast, Barreira et al. (2017) [19] measured higher CF than the one calculated in the present study. This could be attributed to the fact that the *Salicornia* plants used for the analyses were more mature than the ones analyzed in the current study. These inferences imply the importance of the growth stage selected for the analysis of the nutritional properties of halophytes. Young and fresh *Salicornia* tips are much more preferable for food use compared to plants at an advanced vegetative stage [23]. Finally, a higher ash content in halophytic plants compared to other edible crops reflects higher mineral retention due to the saline environment in which they grow [71]. *S. bigelovii* belongs to the group of halophytes that have a tremendous capacity for of inorganic ions storage and water [70]. Higher Na^+^ content was observed for shoots at Mubarak Valley compared to the ones at ICBA, although the salinity of the groundwater applied for irrigation was four-fold less in the former region. This finding could be attributed to the factors explained in Section 4.2. that contributed to retaining more salts in the soil at Mubarak Valley compared to ICBA. In addition, the use of bubblers to irrigate *S. bigelovii* plants at ICBA created the flooding effect facilitating the leaching of salts deeper in the soil (along with the drainage system in place), whereas the drippers installed at Mubarak Valley applied the water more slowly retaining the salts in the upper soil zone where the root system grew. In contrast to Na^+^, the content of ions K^+^, Ca^2+^, Mg^2+^, Zn^2+^ and P^3−^ at Mubarak Valley was much lower than the ones measured at ICBA. These results justify why Na^+^/K^+^ ratio was higher in Mubarak Valley compared to ICBA since Na^+^ and K^+^ content increased and decreased, respectively, at the former location. A similar decline in the ions content with increasing Na^+^ availability was observed for other halophytic species such as *Tetragonia tetragonioides* (New Zealand spinach) [72] and *Cakile maritima* (sea rocket) [66]. Halophytic plants are characterized by an effective nutrient-uptake system that enables them to compartmentalize large amounts of Na^+^ in their vacuoles to lower the osmotic potential and overcome the salinity stress [21,72]. *S. bigelovii* as euhalophyte deals very effectively with salts since the transporters involved in vacuolar Na^+^ accumulation increase almost three times at higher salinity levels corroborating its potent salt adaptation features [73]. VIT B1 and B2 were in low concentrations in both locations; however, VIT C content was high at ICBA compared to Mubarak Valley probably due to the higher salinity of the water applied. VIT C content measured for the shoots at ICBA was higher than the one measured by Lu et al. (2010) [22]. That could be attributed to the younger vegetative stage selected for the analysis in the latter study. 

Considerable diversity of amino acids was observed for shoots in both locations with Glu being predominant among the rest. The average TAA from all genotypes was 862.7 and 729.3 mg/100 g for shoots at ICBA and Mubarak Valley, respectively. Although the average values were lower than 1086 mg/100 g [22], some *S. bigelovii* genotypes grown at ICBA demonstrated TAA higher than 1110 mg/100 g (ICBA-2 and ICBA-10). Based on the results obtained in this study, the shoots contained substantial amounts of essential amino acids, leveraging the value of *S. bigelovii* as a valuable food source.

The fatty acids in the shoots were characterized by a high degree of unsaturation. Actually, the USFA for the shoots at Mubarak Valley ranged between 56.6 and 68.7% percent (Table A6). These findings coincide with other studies on *S. bigelovii* [22], *S. europaea* [74] and *Sarcocornia ambigua* [75] where a high percentage of USFA was measured in their shoots. LA (C18:2 ω6) and ALA (C18:3 ω3) were the main contributors to the USFA composition. The concentrations of these two PUFA were higher in our study compared to the ones measured in the other four Salicorniaceae species *Arthrocnemum macrostachyum*, *S. ramosissima*, *Sarcocornia perennis alpini* and *Sarcocornia perennis perennis* [18]. 

### 4.6. Assessment of the Nutritional Profile of S. bigelovii Seeds

The order of the parameters in the proximate composition of the seeds collected at ICBA and Mubarak Valley was CHO > FAT > PS > CF > Ash > WC and CHO> PS> FAT > CF > Ash >WC, respectively. CHO was the most abundant component in the seeds in both locations and its content was much higher for the genotypes grown at ICBA compared to Mubarak Valley, probably due to the higher level of water salinity applied. CHO concentration in quinoa seeds was affected by the imposed salinity level and it increased up to 200 mM NaCl [76]. Halophytes accumulate high levels of osmolytes, such as CHO, during seed formation to ensure good germination under high saline conditions [77,78]. The genotypes tested in the current study have been under evaluation in increased salinities (between 20–55 dS/m) at ICBA for more than six years which means that they have developed efficient mechanisms of allocating resources from shoots to flowers and then to developing seeds compared to Mubarak Valley where the genotypes are evaluated for the first time. Based on the level of the salt stress applied, a decrease in CHO might be accompanied by an increase in PS as measured in lupin [79]. A similar observation was made for the increased PS of the seeds collected at Mubarak Valley where the CHO content was low. 

Ash was higher for the seeds produced at Mubarak Valley compared to ICBA, probably because the contents of most of the minerals (K^+^, Mg^2+^, Fe^2+^, Mn^2+^, Zn^2+^) were higher in the former region. In contrast, Na^+^, Ca^2+^, and P^3−^ contents were higher for the seeds collected at ICBA. These findings may be due to the higher activity of vacuolar Na^+^/H antiporters, Ca^2+^/H^+^ antiporters and K^+^ transporters in each case which play a critical role in maintaining ion homeostasis [14,78]. Inorganic ions also improve the imbibition process during halophytic seed germination [78]. Na^+^ content in seeds at ICBA was higher than the Na^+^ content in shoots of four out of six genotypes, whereas Na^+^ content in seeds at Mubarak Valley was much lower than the Na^+^ content in shoots of all genotypes. *S. bigelovii* as dicotyledonous halophyte is characterized by an efficient mechanism of Na^+^ compartmentalization in the vacuoles in the shoots and in the seeds coat [77,80] that renders it a good candidate halophytic crop for commercial production in saline environments [12,81]. All micronutrients except Na^+^ were in much higher concentrations in seeds compared to shoots in both locations for all genotypes. The germplasm evaluated in this study was capable to accumulate considerable ion content reiterating the high salt tolerance of this species [10]. Increased microelements (Fe^+2^, Mn^+2^, Zn^+2^) content observed in the seeds collected from Mubarak Valley also strengthens its euhalophytic nature [78]. 

Higher PS for the seeds collected at Mubarak Valley may be correlated with higher K^+^ concentrations that are required for PS synthesis. However, K^+^ is disrupted and damaged in the presence of Na^+^ [80]. Thus, high PS measured in seeds is mainly attributed to the higher content of the essential and non-essential amino acids which increased more than ten-fold and eighteen-fold in the seeds compared to shoots at ICBA and Mubarak Valley, respectively. Similarly to shoots, Glu was the amino acid with the highest concentration in both locations followed by Asp, Cys and Gly. Similar findings were observed for Glu and Asp in two salt marsh grasses *Aeluropus lagopoides* and *Sporobolus madraspatanus* [82] and in *Kosteletzkya virginica* [83]; for Cys in *Suaeda salsa* [84]; and for Glu, Asp and Cys in *Batis maritima* [85]. Glu and Asp protect the cell membranes [82], whereas Cys functions as an osmoprotectant acting as sulphur donor for Met and as a Reactive Oxygen Species (ROS) scavenger during salt stress [84]. 

*S. bigelovii* seeds are characterized by high PS and oil contents comparable to other oilseed crops, such as safflower [81]. Anwar et al. (2002) [28] measured 29.7 and 33.1 percent of oil and protein, respectively, of *S. bigelovii* seeds similar to safflower seeds. These values were higher compared to the ones detected in the current study, probably because the plants were collected from coastal areas in Karachi Pakistan where they were naturally wetted with seawater. In our study, the salinity of the water applied in both locations was much lower than seawater salt content which might have affected the protein and fatty acids composition. The oil content measured in the same genotypes when irrigated with seawater (EC_w_ > 39 dS/m) was higher and reached 39.4% (ICBA-9) [56]. Salinity stress does not only affect the oil content but also the fatty acids composition [86,87,88]. The oil is rich in USFA with LA (C18:2 ω6) being the most abundant fatty acid followed by OA (C18:1 ω9). This is on par with other studies conducted on *S. bigelovii* seeds [28,89]. The low content of ALA (C18:3 ω3) less than 2 percent renders the oil more resistant to oxidation [69]. The USFA/SAFA ratio is used to evaluate the nutritional value of the oil [89]. In our study, the ratio ranged between 3 and 4 which constitute high values compared to other commercial oils. The oil from *S. bigelovii* seeds can be edible since it has nutlike taste and texture similar to olive oil making it pleasant for consumption [81]. The OA (C18:1 ω9)/LA (C18:2 ω6) ratio is related to the shelf life of the oil product [90]. The higher the ratio is, the longer the shelf-life is. In our experiment, this ratio was higher for the seeds collected at ICBA compared to Mubarak Valley. 

Increased USFA content was observed for shoots similarly to seeds at Mubarak Valley; however, the LA (C18:2 ω6) significantly reduced and ALA (C18:3 ω3) content increased. Thus, the oil is less stable due to fast oxidation. However, the high USFA content gives leverage to the quality of the shoots as a vegetable [12]. Leaves and shoots of wild edible plants rich in LA (C18:2 ω6) and ALA (C18:3 ω3) can improve blood functions and enhance human health [91]. 

### 4.7. Differences in S. bigelovii Genotypes in Terms of Performance and Nutritional Composition 

Agronomic parameters and nutritional composition showed strong genotypic responses to salinity of the evaluated germplasm corroborating the rich genetic variation in *S. bigelovii* with respect to salt stress. This variation facilitates breeders when looking into improving the adaptation of the species to diverse environments. Overall, all genotypes were capable to accumulate high levels of ions, strengthening the high salinity tolerance of *S. bigelovii* even at elevated salinities. ICBA-8 produced the highest GBY and was in the top three genotypes for SY at ICBA trials. The opposite was observed for ICBA-10 which produced the highest SY and in the top three genotypes for GBY at the same location. ICBA-10 maintained its preponderance at Mubarak Valley for both GBY and SY with the lowest number of plants per m^2^. ICBA-8 also yielded more seed compared to the rest of the genotypes at the latter location; however, it was ranked four for GBY. ICBA-10 was overall good in ion accumulation, Pro production and TAA content in both locations. These capacities gave ICBA-10 leverage to overcome the elevated salt stress at ICBA trials and also grow well at the new location. Based on PCA for the nutritional traits in shoots, ICBA-3 was consistently good in CHO; ICBA-4 in VIT C; ICBA-8 for GBY; and ICBA-10 for Pro and Zn^2+^. Regarding the PCA for the nutritional traits in seeds, ICBA-3 was consistently good for Zn^2+^ and SAFA; ICBA-4 for the OA (C18:1 ω9)/LA (C18:2 ω6); and ICBA-10 for USFA/SAFA ratio and USFA. Thus, genotypes ICBA-3, ICBA-4 and ICBA-10 demonstrated consistency in nutritional traits.

## 5. Conclusions

This study evaluated the agronomic traits and nutritional composition of six *S. bigelovii* genotypes for food use in the United Arab Emirates and, for the first time, at the Red Sea Governorate in Egypt, using saline groundwater for irrigation. ICBA-10 performed well in both locations and yielded both high green biomass and seed. ICBA-10 was overall also good in ion accumulation, Pro production and TAA content in both locations. Our results indicated that the silt loam texture and the drip irrigation system, along with a lack of a drainage system and leaching fraction, might have contributed to the gradual accumulation of salts in the soil at Mubarak Valley at the end of the experiment higher than ICBA. Apart from the agronomic parameters, salinity also affected the ion accumulation, the amino acids and the fatty acids content for both shoots and seeds, whereas the proximate composition was affected to a lesser extent. Our findings on the high unsaturated fatty acids content corroborate the nutritional value of *S. bigelovii* oil. Due to its euhalophyte nature, *S. bigelovii* is a valuable source of minerals, amino acids and antioxidants that render it the most promising salt-loving plant for food use. This study also increased our knowledge of the intraspecific variability of *S. bigelovii* that could facilitate the breeders to improve the adaptation of the species in saline, hot and dry conditions. Since the experiments were conducted in one season, future experimental work is needed across two locations that appropriately represent the agro-ecological conditions, cropping systems and crop management. In such short-term trials, the primary focus was an initial orientation on how and which genotype out of the six selected ones could perform well in the new location considering the local prevailing environmental factors and their interactions with the genotypes tested. However, the repeatability of the results on a larger scale needs to be investigated. 

## Figures and Tables

**Table 1:** Details of the *S. bigelovii* experiments implemented at ICBA in Dubai in the United Arab Emirates and Mubarak Valley in Marsa Alam in Egypt.

Experiments on *Salicornia bigelovii*	ICBA—UAE	Mubarak Valley—Egypt
Irrigation system	Bubblers	Drippers
Experimental design	RCBD	3 lines per genotype= 18 lines in total
Sowing date	15th of November 2019	20 December 2019
Plant-to-plant spacing	Continuous sowing	20 cm
Compost added	Yes	No
*S. bigelovii* Seedlings’ émergence	19 and 48 DAS * (4 December 2019 and 2 January 2020 respectively)	21 and 49 DAS (9 January and 7 February 2020 respectively)
Number of plants	8 quadrats × 1 measurement per quadrat (1 m^2^)= 8 measurements per genotype in total	1 measurement per row (2 m^2^) × 3 rows = 3 measurements per genotype in total
Plant height (cm)	7 June 2020 8 quadrats × 3 plants per quadrat (1 m^2^)= 24 measurements per genotype in total	10 June 2020 3 plants per row (2 m^2^) × 3 rows = 9 measurements per genotype in total
Green biomass yield (GBY) (kgm^−2^)	7 June 2020 8 quadrats × 1 measurement per quadrat (1 m^2^)= 8 measurements per genotype in total	10 June 2020 1 measurement per row (2 m^2^) × 3 rows= 3 measurements per genotype in total
Seed yield (SY) (gm^−2^)	Late August 8 quadrats × 1 measurement per quadrat (1 m^2^)= 8 measurements per genotype in total	Early September 1 measurement per row (2 m^2^) × 3 rows= 3 measurements per genotype in total

* DAS = Days after sowing.

**Table 2:** Monthly average of daily temperature (Tmin and Tmax), relative humidity (RH) and rainfall in Dubai and Mubarak Valley (MV) over the period 2000 and 2020. Data source: ERA5.

	Tmin		Tmax		RH		Rainfall	
	Dubai	MV	Dubai	MV	Dubai	MV	Dubai	MV
Jan.	13.6	13.5	23.9	21.2	62.7	49.8	10.3	1.4
Feb.	14.4	14.5	26.2	22.8	57.5	45.1	9.3	0.2
Mar.	16.7	17.1	29.9	25.5	52.0	39.7	11.4	0.3
Apr.	20.5	20.4	35.2	28.7	41.6	33.5	4.8	0.1
May.	23.8	24.2	40.1	32.1	36.9	29.6	0.8	0.2
Jun.	26.4	27.0	42.1	34.4	41.7	27.9	0.2	0.0
Jul.	29.3	28.2	43.3	35.6	41.2	30.8	0.8	0.0
Aug.	29.4	28.7	43.2	35.9	41.6	33.0	1.5	0.0
Sep.	26.8	26.8	40.7	33.9	47.2	37.9	0.5	0.0
Oct.	23.3	23.5	36.6	30.7	51.7	47.1	1.1	1.5
Nov.	19.3	19.3	30.5	26.4	56.1	53.1	3.9	0.8
Dec.	15.5	15.6	25.9	22.8	62.8	51.8	9.2	1.2

**Table 3:** Abbreviations and units of the nutritional parameters analyzed for *Salicornia bigelovii* shoots and seeds collected at ICBA and Mubarak Valley.

Parameters	
Proximate Composition Units: g/100 g	Water content (WC); Fat (FAT); Protein (PS); Crude Fiber (CF); Carbohydrates (CHO); Total Ash content (Ash)
Micronutrients Units: mg/100 g	Sodium (Na^+^); Potassium (K^+^); Magnesium (Mg^2+^); Manganese (Mn^2+^); Calcium (Ca^2+^); Phosphorus (P^3−^); Iron (Fe^2+^); Zinc (Zn^2+^); Vitamin C (VIT C); Vitamin B1 (VIT B1); Vitamin B2 (VIT B2)
Amino acids Units: mg/100 g	**Essential amino acids:** Histidine (His); Isoleucine (Ile); Leucine (Leu); Lysine (Lys); Methionine (Met); Phenylalanine (Phe); Threonine (Thr); Valine (Val)
	**Non-Essential amino acids:** Alanine (Ala); Arginine (Arg); Aspartic acid (Asp); Cysteine (Cys); Glutamic acid (Glu); Glycine (Gly); Proline (Pro); Serine (Ser); Tyrosine (Tyr)
Fatty Acids (%) *	**Mono-unsaturated fatty acids (MUFA*):*** Myristoleic acid (C14:1); Pentadecenoic acid (cis-10) (C15:1 ω6); Palmitoleic acid (C16:1 ω7); Trans-palmitoleic acid (C16:1 ω9); Oleic acid (OA) (C18:1 ω9); Vaccenic acid (C18:1 ω7); Gadoleic acid (C20:1); Gondoic acid (C20:1 ω9); Erucic acid (C22:1); Nervonic acid (C24:1) **Poly–unsaturated fatty acids (PUFA):** Linoleic acid (LA) (C18:2 ω6); A-Linolenic acid (ALA) (C18:3 ω3); EPA (C20:5 ω3); Eicosadienoic acid (C20:1 ω7); DHA (C22:6 ω3); Tetracosahexaenoic (C24:6); Pentacosatrienoic (C25:3) **Saturated fatty acids (SAFA):** Capric acid (C10:0); Lauric acid (C12:0); Tridecanoic acid (C13:0); Myristic acid (MA) (C14:0); Pentadecenoic acid (C15:0); Palmitic acid (PA) (C16:0); Heptadecanoic acid (C17:0); Stearic acid (SA) (C18:0); Arachidic acid (C20:0); Lignoceric acid (C24:0)

* Fatty acid content was measured for shoots and seeds collected at Mubarak Valley and for seeds only collected at ICBA.

**Table 4:** Chemical analysis of the groundwater at ICBA’s experimental station and Mubarak Valley.

Parameters	Groundwater at ICBA	Groundwater at Mubarak Valley
EC_w_ (dS/m)	26.0	6.6
pH	7.4	7.4
Anions (meq.L^−1^)		
HCO_3_^−^	1.94	2.2
Cl^−^	204.0	54.3
SO_4_^2−^	53.1	8.5
NO_3_^−^	37.5	0.1
PO_4_^3−^	<0.01	<1.5
Cations (meq.L^−1^)		
Ca^2+^	41.7	19.5
Mg^2+^	45.0	8.0
Na^+^	210.0	37.3
K^+^	3.3	0.2
NH_4_^+^	1.10	2.45
Micronutrients (meq.L^−1^)		
B	<0.01	0.01
Cu	<0.01	<0.2
Fe	<0.01	0.124
Mn	<0.01	0.083
Zn	<0.01	0.02

**Table 5:** Soil analysis of the experimental plots where *S. bigelovii* was cultivated at ICBA and Mubarak Valley.

Parameters	ICBA’s Experimental Station				Mubarak Valley			
	Before the Experiment		After the Experiment		Before the Experiment		After the Experiment	
	Surface Soil ^1^	Subsurface ^2^	Surface Soil	Subsurface	Surface Soil	Subsurface	Surface Soil	Subsurface
pH	7.4	7.5	7.6	7.7	7.9	7.9	7.7	8.0
EC_e_ (dS/m)	3.5	3.2	12.3	14.5	1.9	2.6	28.2	8.9
Saturation Percent (SP%)	26.4	23.4	23.3	21.7	24.0	25.0	25.0	23.0
Anions (meq.L^−1^)								
SO_4_^2−^	77.6	50.2	89.0	54.0	2.0	3.4	109.7	53.1
Cl^−^	98.0	53.4	278.0	126.0	35.1	27.8	211.3	49.2
HCO_3_^−^	2.1	1.5	2.3	1.4	0.9	0.9	2.4	1.7
Cations (meq.L^−1^)								
Na^+^	111.1	54.7	246.8	117.3	19.1	16.9	37.2	31.7
K^+^	5.6	5.6	6.2	5.2	0.4	0.5	2.4	1.7
Mg^2+^	22.6	6.7	44.2	26.2	8.5	5.3	82.0	22.8
Ca^2+^	65.4	27.7	77.0	32.7	10.0	9.4	62.1	37.9
Available nutrients (mg.kg^−1^ soil)								
N	1.1	0.6	2.4	3.0	144.0	103.5	81.0	67.0
K	36.5	28.4	58.9	47.0	165.6	96.8	107.0	104.5
P	8.7	3.4	12.1	7.7	8.0	5.5	0.0	0.0
Cu	1.2	0.8	2.4	3.0	0.1	0.2	0.1	0.1
Fe	1.5	1.3	6.7	2.8	1.0	0.7	0.6	0.7
Mn	0.5	0.4	1.1	0.5	1.8	0.5	0.3	0.3
Zn	0.5	0.6	0.8	0.7	0.3	0.5	0.2	0.2
Soil texture	Sand				Silt Loam			
Sand (%)	96.5				38.5			
Silt (%)	2.4				53.0			
Clay (%)	1.1				8.5			

^1^ Top surface soil: 0–15 cm ^2^ Subsurface soil: 15–45 cm.

**Table 6:** Growth and yield parameters (mean values ± S.E.) of six *Salicornia bigelovii* genotypes evaluated at ICBA and Mubarak Valley. Values having the same letter are not significantly different (*p* < 0.05, *p* < 0.001). LSD multiple comparison test was conducted per trait per location.

ICBA				
*Salicornia bigelovii* genotypes	Number of plants per m^2^	Plant height (cm)	Green biomass (kgm^−2^)	Seed yield (gm^−2^)
ICBA-2	55 ± 16.4 a	50.5 ± 1.9 a	9.5 ± 1.3 ab	69.4 ± 13.5 a
ICBA-3	62 ± 16.4 a	52.1 ± 1.9 a	10.9 ± 1.3 ab	67.4 ± 14.2 a
ICBA-4	50 ± 16.4 a	48.7 ± 1.9 a	9.2 ± 1.3 ab	96.2 ± 14.2 a
ICBA-8	59 ± 16.4 a	53.4 ± 1.9 a	12.2 ± 1.3 b	94.7 ± 14.2 a
ICBA-9	39 ± 16.4 a	48.1 ± 1.9 a	6.2 ± 1.3 a	58.7 ± 14.8 a
ICBA-10	23 ± 16.4 a	52.2 ± 1.9 a	10.9 ± 1.3 ab	116.3 ± 14.2 a
Overall mean	50.9	50.8	9.81	83.77
F-test for genotypes	*NS	NS	*	NS
Mubarak Valley				
ICBA-2	38 ± 1 a	55.5 ± 0.5 bc	7.4 ± 0.4 ab	62.9 ± 5.6 c
ICBA-3	33 ± 6 a	58.0 ± 1.7 ab	5.4 ± 0.3 bc	68.6 ± 3.6 bc
ICBA-4	26 ± 3 a	53.0 ± 0.6 cd	5.0 ± 0.4 c	85.7 ± 5.0 ab
ICBA-8	38 ± 8 a	50.0 ± 0.6 d	6.2 ± 0.5 abc	91.4 ± 4.6 a
ICBA-9	43 ± 5 a	55.0 ± 0.3 bc	7.6 ± 0.5 a	80.0 ± 2.8 abc
ICBA-10	45 ± 3 a	60.0 ± 1.3 a	7.7 ± 0.5 a	82.9 ± 4.4 ab
Overall mean	37	55.2	6.5	78.6
Ftest for genotypes	NS	**	*	*

*NS: non significant, * *p* < 0.05, ** *p* < 0.001.

## Data Availability

The data will be provided by the corresponding author upon request.

## Supplementary Materials

The following supporting information can be downloaded at: https://www.mdpi.com/article/10.3390/plants11192653/s1, Detailed methodologies for analysis of the chemical composition of *S. bigelovii* shoots and seeds.

## Appendix A

Layouts of the field trials at ICBA in the UAE and Mubarak Valley in Egypt.

## Appendix B

Detailed tables with the nutritional composition of shoots and seeds of six *Salicornia bigelovii* genotypes tested at ICBA in the UAE and Mubarak Valley in Egypt.

**Table A1:** Proximate composition (g/100g) and micronutrients (mg/100g) analyzed for shoots of six *Salicornia bigelovii* genotypes collected at three and a half months after sowing at ICBA’s experimental station and Mubarak Valley. The values are calculated on fresh weight (FW) basis. The average value per parameter from all genotypes was calculated (in bold) for each location.

Locations *Salicornia bigelovii* Genotypes Parameters		ICBA (EC_w_ = 26 dSm^−1^)							Mubarak Valley (EC_w_ = 6.6 dSm^−1^)						
		ICBA-2	ICBA-3	ICBA-4	ICBA-8	ICBA-9	ICBA-10	Average	ICBA-2	ICBA-3	ICBA-4	ICBA-8	ICBA-9	ICBA-10	Average
Proximate composition (g/100g FW)	WC	88.9	89.5	88.0	88.9	85.3	88.6	**88.2**	90.3	89.7	89.5	89.5	90.3	91.4	**90.1**
	FAT	0.2	0.1	0.4	0.1	0.2	0.1	**0.2**	0.2	0.2	0.2	0.2	0.2	0.2	**0.2**
	PS	1.1	0.2	1.1	0.8	0.8	1.3	**0.9**	1.0	1.2	1.2	1.2	1.3	1.5	**1.2**
	CF	0.1	0.3	0.1	0.2	0.2	0.2	**0.2**	0.1	0.1	0.1	0.1	0.1	0.1	**0.1**
	CHO	4.8	5.4	4.9	5.7	9.1	4.7	**5.8**	4.4	4.6	4.9	4.9	4.4	3.8	**4.5**
	Ash	4.9	4.5	5.5	4.3	4.4	5.1	**4.8**	4.1	4.2	4.1	4.1	3.7	3.1	**3.9**
Micronutrients (mg/100g FW)	Na^+^	1668.9	1515.1	1693.6	1331.9	1425.2	1593.1	**1538.0**	2774	3254	3370	3937	3870	3371	**3429.3**
	K^+^	285.4	275.6	318.6	293.1	254.9	262.7	**281.7**	144.0	146.7	171.4	170.3	167.4	175.1	**162.5**
	Na^+^/K^+^	5.8	5.5	5.3	4.5	5.6	6.1	**5.5**	19.3	22.2	19.7	23.1	23.1	19.3	**21.1**
	Ca^2+^	163.3	157.8	178.0	155.6	158.4	144.9	**159.7**	83.3	98.1	99.6	117.4	115.4	112.2	**104.3**
	Mg^2+^	118.9	91.7	120.1	97.7	103.5	97.3	**104.9**	71.5	71.5	75.8	88.0	91.4	76.4	**79.1**
	Fe^2+^	1.8	1.3	1.4	1.3	1.5	1.2	**1.4**	1.3	1.8	1.6	2.0	2.7	1.9	**1.9**
	Mn^2+^	0.3	0.2	0.3	0.2	0.2	0.2	**0.2**	0.2	0.2	0.3	0.4	0.3	0.5	**0.3**
	Zn^2+^	0.7	0.7	0.7	0.9	0.9	1.1	**0.8**	0.2	0.1	0.1	0.2	0.2	0.2	**0.2**
	P^3-^	32.1	31.55	37.6	33.5	24.9	24.8	**30.7**	11.5	12.9	14.2	15.9	14.8	13.9	**13.9**
	Vit C	22.5	15	25	25	15	7.5	**18.3**	0.8	0.2	0.5	0.8	0.7	0.1	**0.5**
	Vit B1	ND	ND	ND	ND	ND	ND		0.05	0.03	0.05	0.06	0.04	0.03	**0.04**
	Vit B2	ND	0.03	0.03	0.05	ND	ND		0.04	0.04	0.04	0.04	0.04	0.05	**0.04**

**Table A2:** Essential and non-essential amino acids (mg/100g) analyzed for shoots of six *Salicornia bigelovii* genotypes collected at three and a half months after sowing at ICBA’s experimental station and Mubarak Valley. The values are calculated on fresh weight (FW) basis. The average value per parameter from all genotypes was calculated (in bold) for each location.

Locations *Salicornia bigelovii* Genotypes Amino Acids (mg/100g FW)		ICBA (EC_w_ = 26 dSm^−1^)							Mubarak Valley (EC_w_ = 6.6 dSm^−1^)						
		ICBA2	ICBA3	ICBA4	ICBA8	ICBA9	ICBA10	Average	ICBA2	ICBA3	ICBA4	ICBA8	ICBA9	ICBA10	Average
Essential Amino acids	His	32.5	17.0	23.1	33.1	17.7	58.5	**30.3**	26	25	28	38	32	26	**29.2**
	Ile	82.0	ND	51.5	14.1	33.0	ND	**45.2**	35	36	42	41	40	42	**39.3**
	Leu	107.0	49.2	79.9	55.1	66.0	84.0	**73.5**	59	61	72	71	70	71	**67.3**
	Lys	99.5	56.4	96.9	76.7	64.3	195.5	**98.2**	38	39	46	47	46	47	**43.8**
	Met	10.0	10.0	10.0	10.0	10.0	30.0	**13.3**	10	10	13	15	14	15	**12.8**
	Phe	87.0	40.2	52.5	24.2	40.8	25.8	**45.1**	39	41	48	47	48	47	**45.0**
	Thr	30.0	40.0	40.0	80.0	70.0	120.0	**63.3**	30	33	42	39	40	38	**37.0**
	Val	112.0	56.3	45.7	33.5	65.1	126.0	**73.1**	40	41	49	47	46	47	**45.0**
Non-Essential Amino acids	Ala	60.0	40.0	100.0	130.0	ND	80.0	**82.0**	40	41	48	48	46	47	**45.0**
	Arg	30.9	30.0	72.2	62.7	43.5	72.0	**51.9**	41	42	50	50	48	47	**46.3**
	Asp	ND	ND	ND	ND	ND	ND		70	69	85	84	81	83	**78.7**
	Cys	ND	ND	ND	ND	ND	ND		12	8	8	14	9	15	**11.0**
	Ser	50.0	30.0	40.0	100.0	20.0	50.0	**48.3**	25	31	41	37	40	35	**34.8**
	Glu	240.0	66.0	114.0	51.9	156.0	219.0	**141.2**	91	96	120	113	112	114	**107.7**
	Gly	40.0	20.0	40.0	ND	10.0	90.0	**40.0**	39	40	47	47	46	46	**44.2**
	Pro	47.0	31.2	37.4	43.3	29.7	98.5	**47.8**	33	33	38	38	39	39	**36.7**
	Tyr	89.0	35.1	51.8	15.8	58.0	19.6	**44.9**	6	5	6	5	5	6	**5.5**
	TAA	1116.9	521.4	854.8	730.3	684.0	1268.9	**862.7**	634	651	783	781	762	765	**729.3**

**Table A3:** Proximate composition (g/100g) and micronutrients (mg/100g) analyzed for seeds of six *Salicornia bigelovii* genotypes collected at the end of the growing season at ICBA’s experimental station and Mubarak Valley. The average value per parameter from all genotypes was calculated (in bold) for each location.

Locations *Salicornia bigelovii* Genotypes Parameters		ICBA (EC_w_ = 26 dSm^−1^)							Mubarak Valley (EC_w_ = 6.6 dSm^−1^)						
		ICBA2	ICBA3	ICBA4	ICBA8	ICBA9	ICBA10	Average	ICBA2	ICBA3	ICBA4	ICBA8	ICBA9	ICBA10	Average
Proximate composition (g/100g)	WC	3.7	2.8	3.5	3.2	3.6	3.3	**3.4**	4.1	4.6	4.3	4.2	3.8	3.5	**4.1**
	FAT	20.9	18.2	19.0	19.5	18.6	18.1	**19.1**	18.7	17.9	20.4	20.7	21.1	21.5	**20.1**
	PS	18.8	18.2	18.2	18.8	15.9	18.4	**18.1**	20.0	18.5	22.5	23.5	25.5	26.5	**22.8**
	CF	8.2	7.3	7.5	9.7	7.2	7.9	**8.0**	9.1	8.3	9.5	9.9	9.6	9.9	**9.4**
	CHO	43.7	50.5	47.2	46.6	49.5	46.7	**47.4**	42.3	44.5	37.0	35.5	34.1	32.1	**37.6**
	Ash	4.7	3.0	4.6	2.2	5.2	5.6	**4.2**	5.8	6.2	6.4	6.2	5.9	6.5	**6.2**
Micronutrients (mg/100g)	Na^+^	1820	1124	1760	820	2004	2192	**1620.0**	1367	1421	1450	1415	1375	1467	**1415.8**
	K^+^	748.0	1026.1	1290.7	1155.5	1024.9	1273.5	**1086.5**	954	1123	1259	1210	1153	1305	**1167.3**
	Na^+^/K^+^	2.4	1.1	1.4	0.7	2.0	1.7	**1.6**	1.4	1.3	1.2	1.2	1.2	1.1	**1.2**
	Ca^2+^	311.9	446.7	459.7	368.1	569.3	622.1	**463.0**	259.8	739.1	239.6	222.3	206.5	315.0	**330.4**
	Mg^2+^	365.9	424.1	476.4	384.5	481.5	521.9	**442.4**	543.5	624.7	548.3	550.7	488.2	523.2	**546.4**
	Fe^2+^	9.1	12.7	9.1	10.2	13.4	15.5	**11.7**	15.7	10.7	7.8	18.8	6.1	15.3	**12.4**
	Mn^2+^	3.0	3.0	3.3	3.3	3.2	3.1	**3.1**	9.6	14.6	9.2	9.8	7.8	9.3	**10.1**
	Zn^2+^	7.0	7.3	8.4	8.0	5.6	7.4	**7.3**	10.0	11.2	12.6	10.4	9.9	9.5	**10.6**
	P^3-^	449.6	477.5	466.4	467.0	399.2	423.7	**447.2**	245	365	340	380	335	410	**345.8**
	Vit C	50	50	50	50	50	50	**50**	ND	ND	ND	ND	ND	ND	
	Vit B1	0.6	0.5	0.9	0.6	0.8	0.7	**0.7**	ND	ND	ND	ND	ND	ND	
	Vit B2	0.4	0.2	0.2	0.2	0.3	0.3	**0.3**	0.7	0.4	1.0	ND	ND	ND	

**Table A4:** Essential and non-essential amino acids (mg/100g) analyzed for seeds of six *Salicornia bigelovii* genotypes collected at the end of the growing season at ICBA’s experimental station and Mubarak Valley. The average value per parameter from all genotypes was calculated (in bold) for each location.

Locations *Salicornia bigelovii* Genotypes Amino Acids (mg/100g)		ICBA (EC_w_ = 26 dSm^−1^)							Mubarak Valley (EC_w_ = 6.6 dSm^−1^)						
		ICBA2	ICBA3	ICBA4	ICBA8	ICBA9	ICBA10	Average	ICBA2	ICBA3	ICBA4	ICBA8	ICBA9	ICBA10	Average
Essential Amino acids	His	350	270	270	320	220	270	**283.3**	290	382	390	308	510	242	**353.7**
	Ile	440	390	380	420	350	400	**396.7**	187	237	267	227	322	205	**240.8**
	Leu	880	730	750	850	680	750	**773.3**	457	591	668	586	884	457	**607.2**
	Lys	490	500	440	520	520	500	**495.0**	490	591	593	514	850	405	**573.8**
	Met	130	110	90	120	70	80	**100.0**	109	141	174	150	247	96	**152.8**
	Phe	690	490	510	630	400	520	**540.0**	44	112	77	123	303	114	**128.8**
	Thr	450	380	380	430	350	390	**396.7**	186	251	255	241	358	181	**245.3**
	Val	530	460	460	510	440	490	**481.7**	228	280	332	275	400	222	**289.5**
Non-Essential Amino acids	Ala	490	430	400	490	440	460	**451.7**	561	1060	797	930	1147	567	**843.7**
	Arg	340	290	290	320	200	300	**290.0**	777	141	1656	124	1936	786	**903.3**
	Asp	1020	940	820	1050	1000	970	**966.7**	980	1304	1422	1246	1922	949	**1303.8**
	Cys	90	100	90	100	80	80	**90.0**	208	1602	1800	1557	1937	1322	**1404.3**
	Ser	830	680	700	780	580	660	**705.0**	666	916	1002	854	1276	645	**893.2**
	Glu	2560	2280	2120	2580	2260	2310	**2351.7**	2032	2788	2973	2525	3831	2031	**2696.7**
	Gly	420	310	320	400	320	310	**346.7**	1550	1982	2247	1978	2723	1479	**1993.2**
	Pro	570	520	490	530	470	510	**515.0**	302	223	427	251	576	281	**343.3**
	Tyr	440	320	320	430	260	310	**346.7**	638	735	590	728	1034	606	**721.8**
	TAA	10720	9200	8830	10480	8640	9310	**9530.0**	9705	13336	15670	12617	20256	10588	**13694.0**

**Table A5:** Fatty acids composition (%) analyzed for seeds of six *Salicornia bigelovii* genotypes at ICBA’s experimental station and at Mubarak Valley in Egypt. The average value per parameter from all genotypes was calculated (in bold) for each location.

**Locations** ***Salicornia bigelovii* Genotypes** **Fatty Acids (%)**	**ICBA (EC_w_ = 26 dSm^−1^)**							**Mubarak Valley (EC_w_ = 6.6 dSm^−1^)**						
	**ICBA2**	**ICBA3**	**ICBA4**	**ICBA8**	**ICBA9**	**ICBA10**	**Average**	**ICBA2**	**ICBA3**	**ICBA4**	**ICBA8**	**ICBA9**	**ICBA10**	**Average**
**Mono-unsaturated**														
Myristoleic acid (C14:1)	ND	ND	ND	ND	ND	ND		1.2	1.1	ND	ND	0.7	ND	
OA (C18:1 ω9)	16.6	19.8	20.5	16.7	15.9	19.5	**18.2**	0.7	9.0	9.2	10.3	11.2	1.2	**6.9**
Palmitoleic acid (C16:1 ω7)	ND	ND	ND	ND	ND	ND		1.2	ND	0.8	ND	ND	0.9	
Gadoleic acid (C20:1)	ND	ND	ND	1.2	ND	ND		ND	ND	ND	ND	ND	ND	
Erucic acid (C22:1)	ND	ND	ND	ND	ND	ND		1.0	ND	ND	0.9	ND	ND	
Nervonic acid (C24:1)	ND	ND	ND	ND	ND	ND		ND	ND	1.4	ND	ND	0.8	
**Poly-unsaturated**														
LA (C18:2 ω6)	57.0	48.4	57.4	56.3	55.9	56.6	**55.3**	55.6	39.1	46.3	46.1	54.9	62.2	**50.7**
ALA (C18:3 ω3)	1.5	1.6	1.6	3.0	2.0	1.5	**1.9**	ND	ND	ND	ND	ND	ND	
EPA (C20:5 ω3)	0.9	1.1	1.1	0.3	1.1	0.8	**0.9**	ND	ND	ND	ND	ND	ND	
DHA (C22:6 ω3)	1.1	1.1	1.1	1.1	1.1	0.8	**1.0**	ND	ND	ND	ND	ND	ND	
Tetracosahexaenoic (C24:6)	ND	ND	ND	ND	ND	ND		1.6	1.0	4.1	2.3	2.2	3.0	
Pentacosatrienoic (C25:3)	ND	ND	ND	ND	ND	ND		1.2	0.9	2.2	1.1	1.0	1.6	
**Saturated**														
Capric acid (C10:0)	ND	ND	ND	ND	ND	ND		2.9	1.1	4.0	1.6	1.4	1.8	**2.1**
Lauric acid (C12:0)	ND	ND	ND	ND	ND	ND		6.6	16.9	0.5	4.8	ND	ND	
MA (C14:0)	0.7	0.5	0.5	0.9	0.7	0.7	**0.7**	3.0	6.9	3.0	3.4	1.5	1.6	**3.2**
PA (C16:0)	14.0	15.4	12.7	12.1	12.7	14.1	**13.5**	23.5	20.8	25.3	25.4	22.7	25.6	**23.9**
Heptadecanoic acid (C17:0)	ND	ND	ND	ND	ND	ND		ND	ND	ND	ND	ND	ND	
SA (C18:0)	6.7	6.6	4.9	5.3	6.3	5.0	**5.8**	2.6	3.2	3.9	4.1	4.4	3.3	**3.6**
Arachidic acid (C20:0)	0.6	1.3	1.1	1.4	0.9	0.8	**1.0**	ND	ND	ND	ND	ND	ND	
Lignoceric acid (C24:0)	1.1	1.2	1.2	1.2	1.1	0.8	**1.1**	ND	ND	ND	ND	ND	ND	
Total unsaturated (USFA)	77.1	72	81.7	78.6	76	79.2	**77.4**	62.5	51.1	64	60.7	70	69.7	**63.0**
Total saturated (SAFA)	23.1	25	20.4	20.9	21.7	21.4	**22.1**	38.6	48.9	36.7	39.3	30	32.3	**37.6**
USFA/SAFA	3.3	2.9	4.0	3.8	3.5	3.7	**3.5**	1.6	1.0	1.7	1.5	2.3	2.2	**1.7**
OA/LA	0.3	0.4	0.4	0.3	0.3	0.3	**0.3**	0.01	0.2	0.2	0.2	0.2	0.02	**0.1**

**Table A6:** Fatty acids composition (in percent) analyzed for shoots and seeds of six *Salicornia bigelovii* genotypes at Mubarak Valley in Egypt. The average value per parameter from all genotypes was calculated (in bold) for each location.

Part of the Plant *Salicornia bigelovii* Genotypes Fatty Acids (%)	Shoots							Seeds						
	ICBA2	ICBA3	ICBA4	ICBA8	ICBA9	ICBA10	Average	ICBA2	ICBA3	ICBA4	ICBA8	ICBA9	ICBA10	Average
**Mono-unsaturated**														
Myristoleic acid (C14:1)	ND	ND	ND	ND	ND	ND		1.2	1.1	ND	ND	0.7	ND	
Pentadecenoic acid (cis-10) (C15:1 ω6)	ND	0.9	ND	ND	ND	ND		ND	ND	ND	ND	ND	ND	
Palmitoleic acid (C16:1 ω7)	ND	0.8	0.5	0.3	0.6	ND		1.2	ND	0.8	ND	ND	0.9	
Trans-palmitoleic acid (C16:1 ω9)	5.8	3.4	6.3	6.5	5.8	4.1	**5.3**	ND	ND	ND	ND	ND	ND	
OA (C18:1 ω9)	3.2	17.7	3.3	2.5	5.0	5.2	**6.1**	0.7	9.0	9.2	10.3	11.2	1.2	**6.9**
Vaccenic acid (C18:1 ω7)	0.5	0.7	0.5	0.4	0.5	0.6	**0.5**	ND	ND	ND	ND	ND	ND	
Gondoic acid (C20:1 ω9)	ND	0.3	ND	ND	ND	ND		ND	ND	ND	ND	ND	ND	
Erucic acid (C22:1)	ND	ND	ND	ND	ND	ND		1.0	ND	ND	0.9	ND	ND	
Nervonic acid (C24:1)	ND	ND	ND	ND	ND	ND		ND	ND	1.4	ND	ND	0.8	
**Poly-unsaturated**														
LA (C18:2 ω6)	25.4	14.8	22.5	24.0	26.0	25.0	**22.9**	55.6	39.1	46.3	46.1	54.9	62.2	**50.7**
ALA (C18:3 ω3)	32.3	17.4	34.3	34.9	30.8	29.1	**29.8**	ND	ND	ND	ND	ND	ND	
Eicosadienoic acid (C20:1 ω7)	ND	0.6	ND	ND	ND	2.4		ND	ND	ND	ND	ND	ND	
Tetracosahexaenoic (C24:6)	ND	ND	ND	ND	ND	ND		1.6	1.0	4.1	2.3	2.2	3.0	**2.4**
Pentacosatrienoic (C25:3)	ND	ND	ND	ND	ND	ND		1.2	0.9	2.2	1.1	1.0	1.6	**1.3**
**Saturated**														
Capric acid (C10:0)	ND	ND	ND	ND	ND	ND		2.9	1.1	4.0	1.6	1.4	1.8	**2.1**
Lauric acid (C12:0)	ND	0.3	0.2	ND	0.1	ND		6.6	16.9	0.5	4.8	ND	ND	
Tridecanoic acid (C13:0)	ND	0.3	0.7	0.5	0.5	ND		ND	ND	ND	ND	ND	ND	
MA (C14:0)	1.0	1.2	1.1	0.9	1.0	0.9	**1.0**	3.0	6.9	3.0	3.4	1.5	1.6	**3.2**
Pentadecenoic acid (C15:0)	0.8	1.7	0.7	0.9	1.2	3.6	**1.5**	ND	ND	ND	ND	ND	ND	
PA (C16:0)	22.9	29.9	21.5	21.0	21.0	21.9	**23.0**	23.5	20.8	25.3	25.4	22.7	25.6	**23.9**
Heptadecanoic acid (C17:0)	2.5	1.6	2.4	2.4	2.3	0.6	**2.0**	ND	ND	ND	ND	ND	ND	
SA (C18:0)	1.7	3.7	1.8	1.6	1.8	2.1	**2.1**	2.6	3.2	3.9	4.1	4.4	3.3	**3.6**
Arachidic acid (C20:0)	0.5	0.7	0.6	0.7	0.7	0.6	**0.6**	ND	ND	ND	ND	ND	ND	
Behenic acid (C22:0)	1.9	1.4	1.6	1.7	1.3	1.2	**1.5**	ND	ND	ND	ND	ND	ND	
Total unsaturated (USFA)	67.2	56.6	67.4	68.6	68.7	66.4	**54.8**	62.5	51.1	64	60.7	70	69.7	**63**
Total saturated (SAFA)	31.3	40.8	30.6	29.7	29.9	30.9	**27.1**	38.6	48.9	36.7	39.3	30	32.3	**37.6**
USFA/SAFA	2.1	1.4	2.2	2.3	2.3	2.1	**2.1**	1.6	1.0	1.7	1.5	2.3	2.2	**1.7**
OA/LA	0.1	1.2	0.2	0.1	0.2	0.2	**0.3**	0.01	0.2	0.2	0.2	0.2	0.02	**0.1**

## Appendix C

Eigenvector values per Principal Component Analysis (PCA) Dimension 1 and 2 (Dim1 and Dim2) for the yield parameters, proximate composition, micronutrients, amino acids and fatty acids for the shoots and seeds of six *S. bigelovii* genotypes at ICBA in the UAE and Mubarak Valley in Egypt.

**Table A7:** Eigenvector values per Principal Component Analysis (PCA) Dimension 1 and 2 (Dim1 and Dim2) for the green biomass yield, proximate composition, micronutrients and amino acids measured for the shoots of six *S. bigelovii* genotypes at the vegetative stage at ICBA’s experimental station in the United Arab Emirates (Figure 4a) and Mubarak Valley in Egypt (Figure 4b). The higher value for each parameter between the two axes is in bold.

*S. bigelovii* Shoots					
Parameters		ICBA		Mubarak Valley	
		PCA Dim1 (41.5%)	PCA Dim2 (29.8%)	PCA Dim1 (51.9%)	PCA Dim2 (22.8%)
Yield parameter	GBY	−0.100	**−0.341**	0.229	**−0.601**
Proximate Composition	FAT	**0.856**	−0.061	0.001	0.001
	PS	0.429	**0.745**	**0.749**	−0.525
	CF	**−0.864**	−0.303	0.001	0.001
	CHO	**−0.390**	−0.186	−0.130	**0.941**
	Ash	**0.713**	0.475	−0.575	**0.811**
Micronutrients	Na+	**0.728**	0.486	**0.870**	0.447
	K^+^	**0.782**	−0.416	**0.914**	−0.090
	Na^+^/K^+^	−0.008	**0.822**	0.381	**0.678**
	Ca^2+^	**0.858**	−0.412	**0.957**	0.109
	Mg^2+^	**0.948**	0.160	**0.798**	0.401
	Fe^2+^	**0.561**	0.077	**0.751**	0.179
	Mn^2+^	**0.981**	0.093	**0.804**	−0.425
	Zn^2+^	**−0.621**	0.567	**0.367**	−0.324
	P^3-^	**0.708**	−0.594	**0.890**	0.417
	VIT C	**0.668**	−0.617	−0.037	**0.578**
Aminoacids	Glu	0.226	**0.900**	**0.841**	0.084
	Pro	−0.219	**0.825**	**0.952**	−0.076
	TAA	0.260	**0.891**	**0.908**	0.120

**Table A8:** Eigenvector values per Principal Component Analysis (PCA) Dimension 1 and 2 (Dim1 and Dim2) of seed yield, proximate composition, micronutrients, amino acids and fatty acids measured for the seeds of all *S. bigelovii* genotypes after harvest at ICBA in the United Arab Emirates (Figure 5a) and Mubarak Valley in Egypt (Figure 5b). The higher value for each parameter between the two axes is in bold.

*S. bigelovii* Seeds					
		ICBA		Egypt	
Parameters		PCA Dim1 (37%)	PCA Dim2 (22.5%)	PCA Dim1 (42.8%)	PCA Dim2 (27.4%)
Yield parameter	SY	**−0.341**	−0.092	**0.707**	0.175
Proximate Composition	FAT	**0.803**	0.422	**0.965**	−0.155
	PS	**0.627**	−0.298	**0.951**	−0.213
	CF	**0.709**	0.078	**0.856**	−0.345
	CHO	**−0.568**	−0.551	**−0.953**	0.185
	Ash	−0.613	**0.647**	**0.227**	−0.040
Micronutrients	Na+	−0.604	**0.655**	**0.199**	−0.090
	K^+^	**−0.595**	−0.338	**0.618**	0.122
	Na^+^/K^+^	−0.106	**0.764**	**−0.781**	−0.019
	Ca^2+^	**−0.927**	0.136	**−0.725**	0.291
	Mg^2+^	**−0.965**	0.078	**−0.869**	0.239
	Fe^2+^	**−0.647**	0.089	−0.249	**−0.713**
	Mn^2+^	**−0.178**	−0.019	**−0.835**	0.257
	Zn^2+^	0.250	**−0.549**	−0.229	**0.600**
	P^3-^	0.532	**−0.784**	**0.360**	0.125
Aminoacids	Asp	**0.536**	0.469	0.435	**0.816**
	Cys	0.666	**−0.730**	0.484	**0.764**
	Glu	**0.871**	0.318	0.399	**0.852**
	Gly	**0.920**	0.360	0.418	**0.857**
	Pro	**0.869**	0.072	**0.672**	0.420
	TAA	**0.936**	0.212	0.515	**0.798**
Fatty acids	C18:1 ω9	−0.421	**−0.664**	0.194	**0.918**
	C18:2 ω6	0.009	**0.725**	0.568	**−0.721**
	USFA	−0.129	**0.305**	**0.890**	−0.342
	SAFA	0.243	**−0.358**	**−0.915**	0.259
	USFA/SAFA	−0.215	**0.247**	**0.897**	−0.264
	C18:1 ω9 / C18:2 ω6	−0.228	**−0.836**	0.083	**0.941**
