# Supplementary material for: Evaluation of Salicornia bigelovii Germplasm for Food Use in Egypt and the United Arab Emirates Based on Agronomic Traits and Nutritional Composition
Source: Plants (Basel). 2022 Oct 9;11(19):2653. doi: 10.3390/plants11192653 (PMC9572425; doi:10.3390/plants11192653)
Supplement: Supplementary file 1 [file plants-11-02653-s001.zip › plants-1808576-supplementary.pdf]

Supplementary material

Detailed methodologies for analysis of the chemical composition of *Salicornia bigelovii* shoots and seeds

The nutritional profile of shoots and seeds of all six *S. bigelovii* genotypes was studied for both locations by analyzing the proximate composition, micronutrients, amino acids and fatty acids content.

Sampling

Sampling was performed randomly in the replications (three plots at ICBA and three rows at Mubarak Valley) and one composite sample of fresh tips from different plants per genotype was prepared for the nutritional analyses in both locations. Similar approach was followed for the preparation of composite samples of seeds from all three replications per genotype at the two locations. Thus, six samples of shoots and six samples of seeds were analyzed in total per location. The fresh tips were cut with scissors approximately three and a half months after sowing; on 3rd of March at ICBA and on 5th of April at Mubarak Valley, whereas seed samples were prepared after the final harvest of the dry biomass for the seeds collection. The samples of the fresh tips and seeds prepared at ICBA were individually placed in plastic bags and transported to the Food Allergens Laboratory, Cyprus (ISO/IEC 17025:2017) (https://www.foodallergenslab.com/#!/en) for analysis. The quantity per sample was 1.5 kg. At Mubarak valley, 2.5 kg per sample of fresh tips was prepared and sent for analysis at the Regional Center for Food and Feed, Agriculture research Center of the Ministry of Agriculture and land Reclamation in Cairo. Seed samples were prepared by removing them through mechanic separation. The detailed protocols for the analysis of the chemical composition are provided below and the detection limits of the equipment used per group of analysis in Table S1 in Supplementary material.

- **Fat content** (Fat) was determined by the Soxhlet extraction method as described by [49].
- **Protein** (PS) was measured by the Kjeldahl method, as described by [49].
- **Water content** (WC) was determined by the method described by [49]
- **Ash content** (Ash) was analyzed after burning the plants in a muffle furnace [49]
- **Crude fiber (CF)** content was determined using the neutral detergent reagent method [21].
- **Total carbohydrate (CHO) content** was estimated by difference between 100 and the sum of the percentages of moisture, protein, total lipid, and ash contents [21].
- **Minerals content**: sodium (Na), potassium (K), magnesium (Mg), manganese (Mn), calcium (Ca), phosphorus (P), iron (Fe) and zinc (Zn), were analyzed using the Inductive Coupled Plasma (ICP) Emission spectrometer according to [50].
- **Vitamins content**: Vitamins C, B1 and B2 were measured based on high-performance liquid chromatography (HPLC) analysis as follows:

    a) Device Specification: Waters 2690 Alliance HPLC system equipped with a Waters 996 photodiode array detector.

b) Standard preparation: Weigh 10mg of 7 water soluble vitamins reference standards (Ascorbic acid, Thiamine HCl, Riboflavin, Nicotinic acid, Nicotinamide, Pyridoxine HCl and Folic acid) in 10ml 0.05 M NaOH, dilute to concentration 100µg/ml and solution was filtered
on 0.22 µm syringe filter then 100 µl was injected.

c) Sample preparation: Five Extracts were prepared with concentration of 50mg/ml, filtered on 0.22 µm syringe filter then 100 µl was injected.

d) HPLC analysis conditions:

- Column Inertsil ODS 3: 4.6x250mm, 5µm
- Mobile phase: (0.85gm Hexane sulphonic acid in 1000 ml water and adjust pH to 3 with orthophosphoric acid): Methanol
- Mode of elution: Gradient
- Flow rate: 1 ml/min
- Temperature: Ambient
- Wavelength: 230 nm

- Amino acids were determined using Sykam Amino Acid Analyzer (Sykam GmbH, Germany) as follows:

a) ***Device Specification:*** Sykam Amino Acid Analyzer (Sykam GmbH, Germany) equipped with Solvent Delivery System S 2100 (Quaternary pump with flow range 0.01 to 10.00 ml/min and maximum pressure up to 400 bar), Autosampler S 5200, Amino Acid Reaction Module S4300 (with built-in dual filter photometer between 440 and 570 nm with constant signal output and signal summary option) and Refrigerated Reagent Organizer S 4130.

b) ***Standard preparation:*** Stock solution contains 18 Amino acids (Aspartic acid, Threonine, Serine, Glutamic acid, Proline, Glycine, Alanine, Cysteine, Valine, Methionine, Isoleucine, Leucine, Tyrosine, Phenylalanine, Histidine, Lysine, Ammonia, Arginine) all amino acids concentrations are 2.5µMol/ml, except Cystine 1.25µMol/ml, then dilute 60 µl in 1.5 ml vial with sample dilution buffer then filtered using 0.22 µm syringe filter then 100 µl was injected.

c) ***Sample preparation:*** 1 gm of each sample was mixed with 5 mL hexane. The mixture was allowed to macerate for 24 hours. Then, the mixture was filtered on whatman no. 1 filter paper and the residue was transferred into a test tube where it was incubated in an oven with 10 mL 6N HCl for 24h at 110°C. After the incubation, the sample was filtered on whatman no. 1 filter paper, evaporated on rotary evaporator and dissolved completely in 2 ml dilution buffer. From this solution, the first dilution was prepared by diluting 100 µL to 1 mL dilution buffer and from which 10µL were further diluted to 1mL dilution buffer, filtered using 0.22 µm syringe filter and 100 µl was injected.

d) ***Instrument parameters:***

- Column: LCA K06/Na
- Mobile phase: Buffer A, Buffer B and Regeneration solution
- Mode of elution: Gradient

- Flow rate: 0.45 ml/min
- Temperature: Gradient 57ºC - 74ºC
- Wavelength: 440 and 570 nm

- Fatty acid composition: The fat extracted from seeds was further analyzed for the fatty acids composition using the gas chromatography method. Identification of each fatty acid was conducted using the equivalent chain lengths and laboratory standards. All the results for the chemical composition of shoots and seeds was expressed on fresh and dry weight basis respectively.

Preparation of fatty acid methyl ester (FAME) was carried out according to [51-52]. A standard mixture of fatty acid methyl esters was used to identify of the peaks by their retention time. The different fatty acid methyl esters (FAMEs) were determined and identified using a gas chromatography (Hewlett Packard 6890) equipped with a flame ionization detector (FID). A HP-5 column (30m, 0.32mm ID, 0.25µm film thickness) [5% diphenyl, 95% dimethyl polysiloxane] was used. The detector and injector temperatures were 280ºC and 220ºC, respectively. Sample size 3 µl, spilt ratio 50:1. Nitrogen was used as carrier gas at a flow rate of 1 ml/min. Oven temperature was programmed as: - Set point (initial temperature) 150ºC for 2 Min. - Rate 10ºC/min to 200ºC. - Rate 5ºC/min to 250ºC and hold for 9 Min. The concentrations of fatty acids were calculated as following equation: Fatty acid% = peak area ÷ overall area of peaks × 100.

Table S1. Methods applied for the nutritional analyses of *Salicornia bigelovii* shoots and seeds along with the detection limits of the equipment used.

Parameters		Detection limits	Methodology
Proximate Composition (g/100g)	Water content (WC)	-	Calculated
	Fat (FAT)	0.1	Soxhlet Inhouse Method
	Protein (PS)	0.1	Kjeldahl method
	Crude Fiber (CF)		Neutral detergent reagent method
	Carbohydrates (CHO)		Calculated
	Total Ash content (Ash)	0.01	AOAC 1990
Micronutrients (mg/100g)	Sodium (Na^{+})<br>Potassium (K^{+})<br>Magnesium (Mg^{2+})<br>Manganese (Mn^{2+})<br>Calcium (Ca^{2+})<br>Phosphorus (P^{3-})<br>Iron (Fe^{2+})<br>Zinc (Zn^{2+})	0.01	ICP-OES CYS EN 16943:2017<br>Atomic absorption
	Vitamins	0.1 (UAE)<br>0.01 (Egypt)	HPLC
Aminoacids (mg/100g)		10 (UAE)<br>0.5 (Egypt)	LC-MS/MS<br>Sykam Amino-Acid Analyzer (Sykam GmbH, Germany)
Fatty Acids (%)		0.01	GLC EEC Regulation 2568/91

	GC-MS
